# Supplementary figures and images for: Phase separation of a PKA type I regulatory subunit regulates β-cell function through cAMP compartmentalization
Source: PLoS Biol. 2025 Jul 24;23(7):e3003262. doi: 10.1371/journal.pbio.3003262 (PMC12289088; doi:10.1371/journal.pbio.3003262)

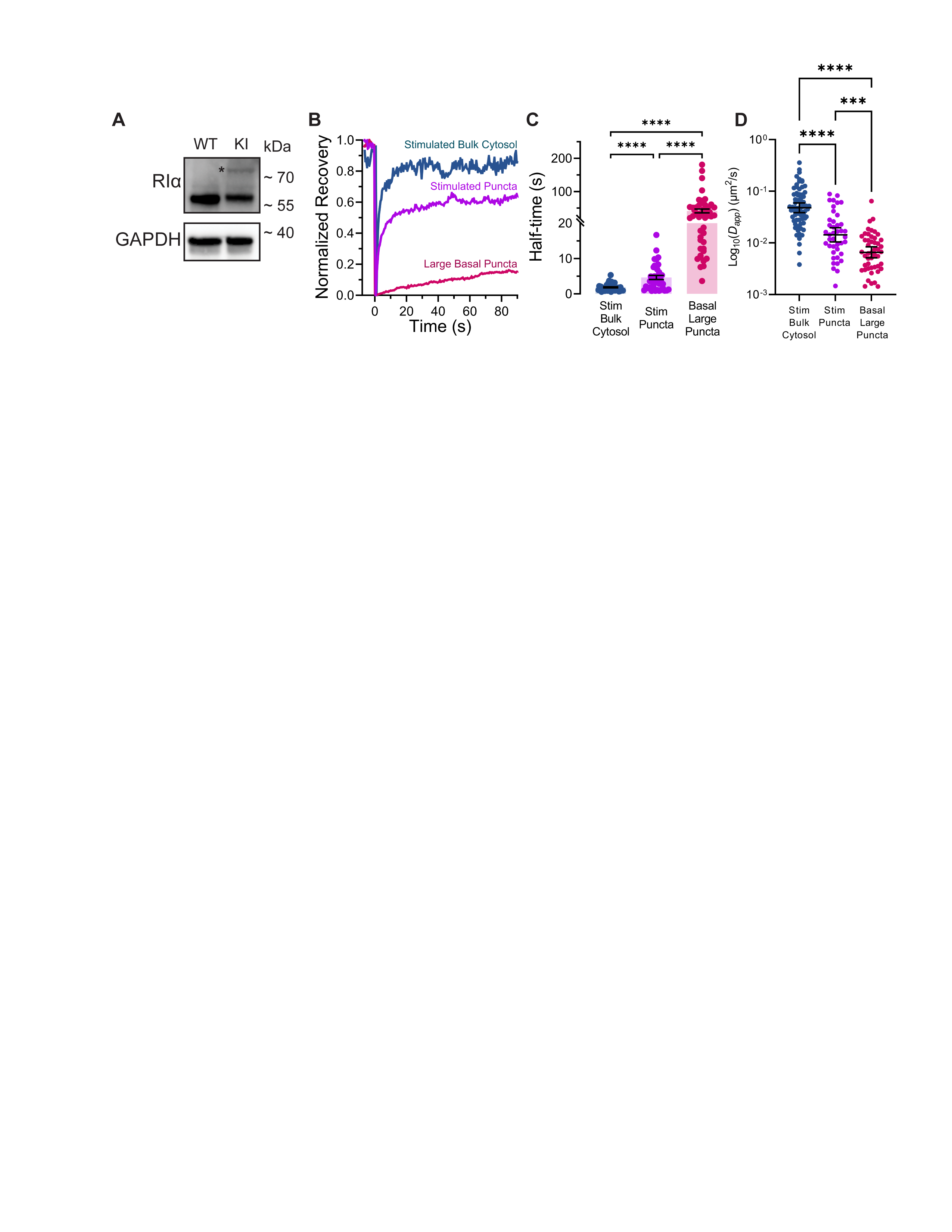

Supplement: S1 Fig — (A) Western blot analysis confirming successful CRISPR-mediated homology-directed repair insertion of GFP2 into the RIα locus in MIN6 β cells (KI). Asterisk indicates GFP2-tagged RIα. (B) Representative time courses of normalized recovery of fluorescence intensity after photobleaching of the indicated regions. (C) Quantification of the recovery half-time after photobleaching of stimulated bulk cytosol (n = 44 regions), stimulated puncta (n = 41 puncta), and basal large puncta (n = 48 puncta). ****P = 5.89 × 10^−5^, stimulated bulk cytosol versus stimulated puncta; ****P = 1.80 × 10^−8^, stimulated bulk cytosol versus basal large puncta; and ****P = 1.05 × 10^−7^, stimulated puncta versus basal large puncta; Brown-Forsythe and Welch one-way ANOVA with Dunnett’s T3 multiple comparisons test. Error bars indicate mean ± SEM. (D) Quantification of apparent diffusion coefficients (Dapp) across different cellular compartments. The graph shows Log10(Dapp) values in μm2/s for stimulated bulk cytosol (n = 68), stimulated puncta (n = 42), and basal large puncta (n = 48). Statistical significance is indicated by asterisks (****P < 0.0001, ***P < 0.001). Error bars indicate mean ± 95% CI, and statistics indicated by Brown-Forsythe and Welch ANOVA followed by Dunnett’s T3 multiple comparisons test. The data underlying this figure can be found in S1 Data and S1 Raw Images. (TIF) [file pbio.3003262.s001.tif]

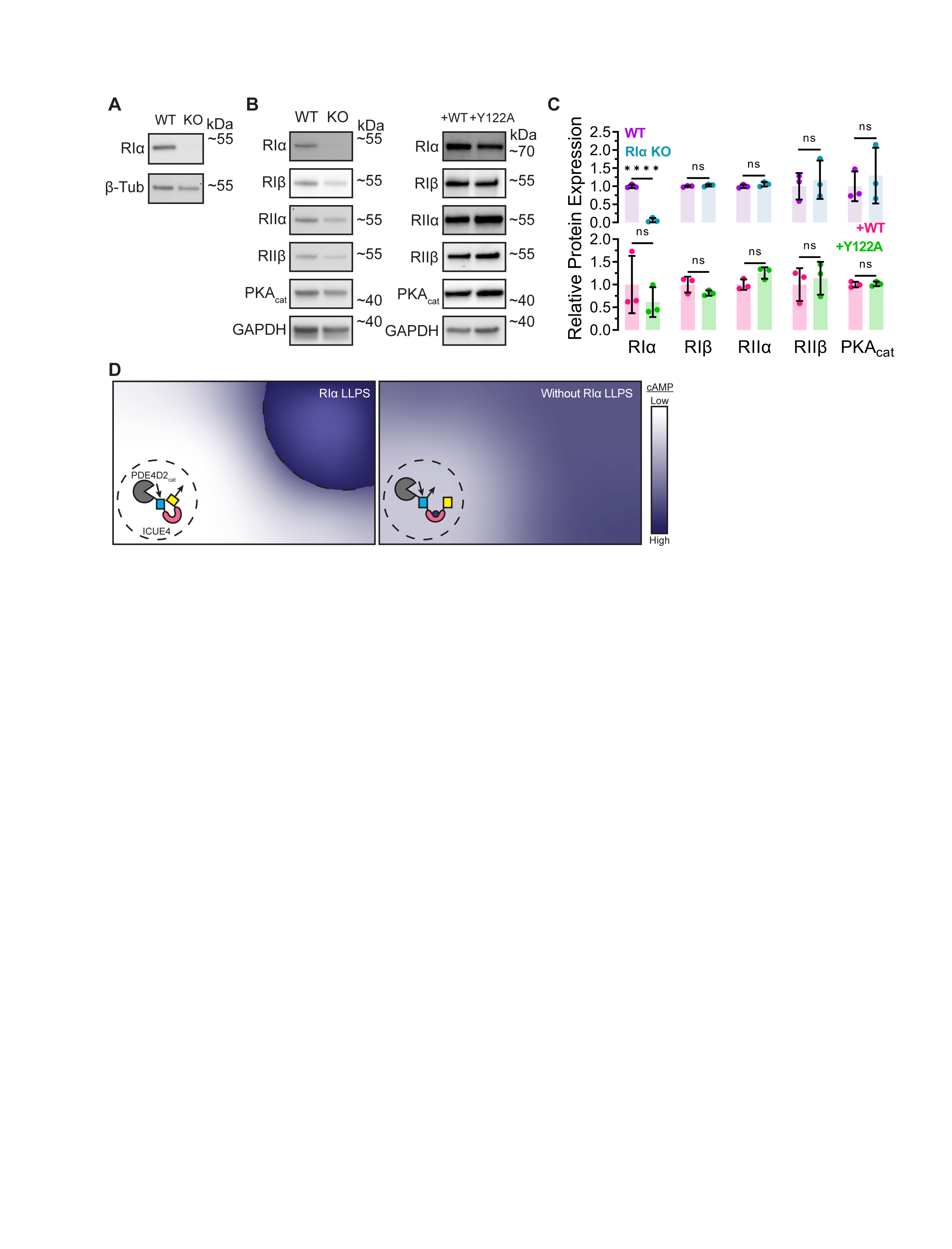

Supplement: S2 Fig — (A) Western blot results showing knockout of RIα in CRISPR/Cas9-engineered MIN6 β cell line. (B) Western blot analysis of PKA subunit expression in WT and RIα-null MIN6 β cells (left), and in RIα-null cells expressing either RIαWT or RIαY122A (right). Representative blots from three independent experiments. (C) Quantification of western blot data comparing WT versus RIα-null MIN6 β cells (top) and RIαWT versus RIαY122A expression (bottom). n = 3 experiments, ****P < 0.0001, unpaired two-tailed Student t test. Data represent mean ± SD. (D) Schematic overview of the cAMP compartmentalization assay. The FRET-based cAMP indicator ICUE4 is tethered to the PDE4D2cat to specifically probe cAMP accumulation in the vicinity of PDE4D2. The data underlying this figure can be found in S1 Data and S1 Raw Images. (TIF) [file pbio.3003262.s002.tif]

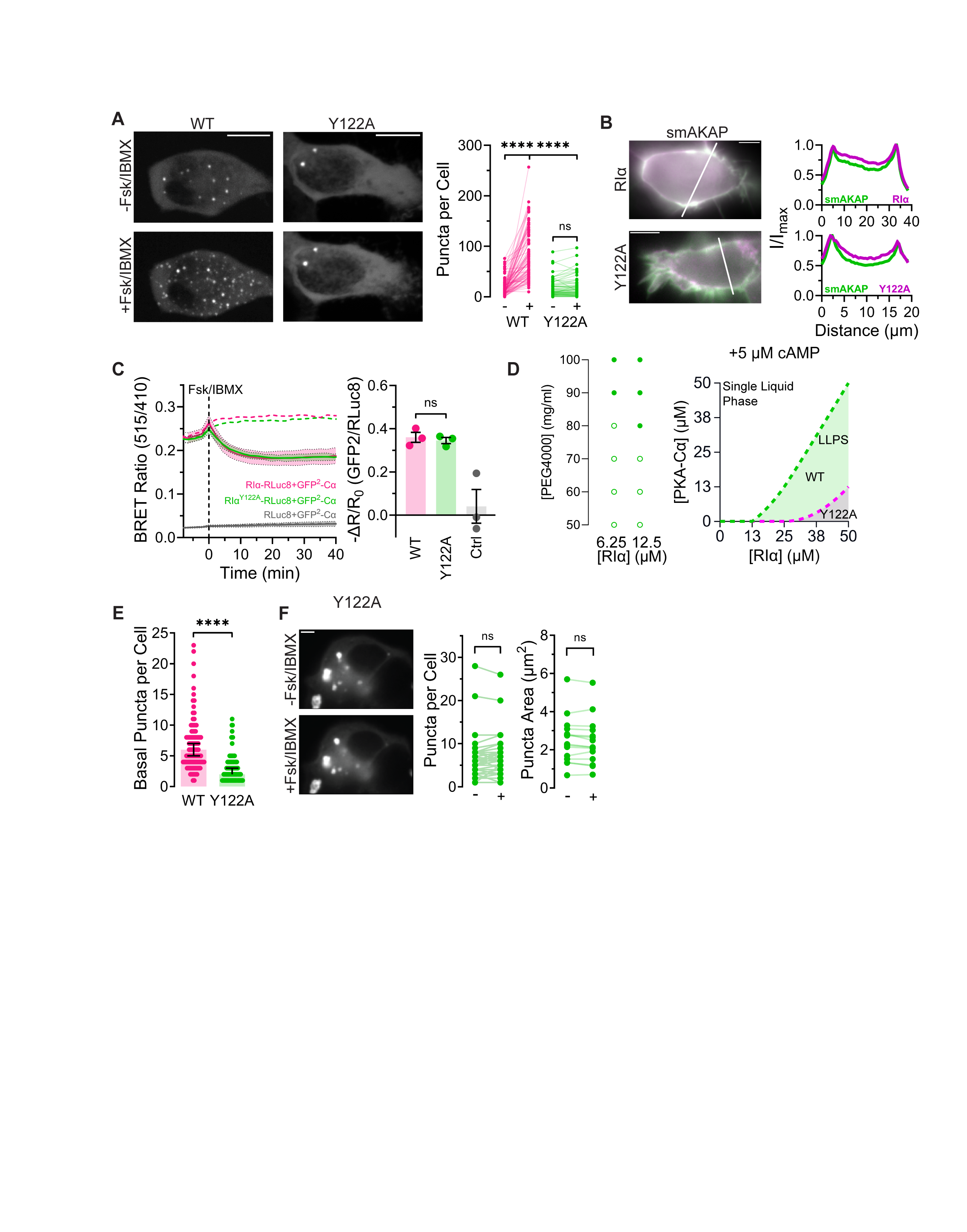

Supplement: S3 Fig — (A) Representative maximum intensity projections from confocal Z-stacks showing GFP expression (left) and quantification of puncta per cell (right) from HEK293T cells co-expressing Cα-mCherry plus GFP2-tagged RIαWT (WT, n = 90 cells) or RIαY122A (Y122A, n = 59 cells) before (−) and after (+) Fsk and IBMX stimulation. ****P < 1 × 10^−15^; 2-way ANOVA with Šídák’s multiple comparisons test. (B) Representative fluorescence images and line-intensity profiles of the indicated regions in HEK293T cells expressing smAKAP-EGFP with mRuby2-tagged RIαWT or RIαY122A. (C) BRET assay time-course (left) and quantification of Fsk-stimulated change (right) in the GFP2/RLuc8 emission ratio in HEK293T cells co-expressing GFP2-Cα plus RLuc8 fused to either RIαWT (n = 3 experiments) or RIαY122A (n = 3 experiments). Untethered RLuc8 co-expressed with GFP2-Cα is used as a control. P = 0.61, unpaired, two-tailed Student t test. Solid lines indicate the mean, and shaded areas indicate SEM. (D) Critical saturation concentration (C-sat) curves of RIαY122A. In vitro phase diagram of RIαY122A vs. PEG 4000 (Left). Filled circles represent droplets, and empty circles represent no droplets. C-sat curves showing PKA-Cα concentration-dependent LLPS behavior of RIαWT (green) and RIαY122A (magenta) (Right). Dotted lines indicate the boundary between one-phase and two-phase regions (shaded areas). RIαWT data were modified from our previous work [10]. (E) Quantification showing basal puncta number per cell in RIα-null MIN6 β cells expressing either mRuby2-tagged RIαWT (WT; n = 98 cells) or RIαY122A (Y122A; n = 103 cells). ****P = 5.47 × 10^−12^ (WT vs. Y122A); unpaired Komogorov-Smirnov test. Error bars in summary quantification indicate median ± 95% CI. (F) Representative images (left) and quantification of RIαY122A puncta number (n = 53 cells) (middle) and area (for pre-existing puncta) (n = 15 puncta) (right) in RIα-null MIN6 β cells. ns, P = 0.17 for puncta number per cell; ns, P = 0.12 for pre-existing [file pbio.3003262.s003.tif]

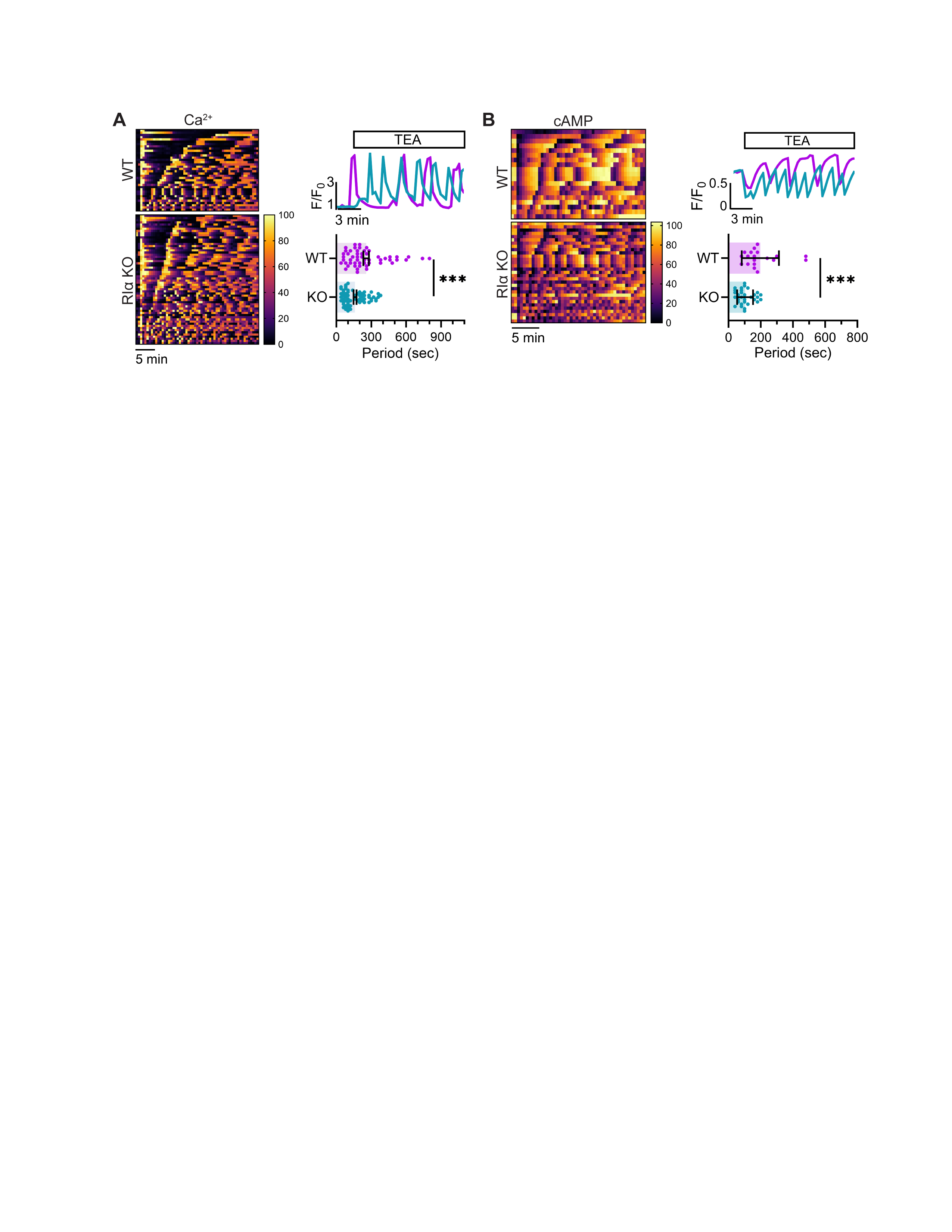

Supplement: S4 Fig — (A) Heatmap (left) showing Ca2+ oscillations in individual WT or RIα-null (RIα KO) MIN6 β cells (each row) plotted as normalized RCaMP intensity. Cells were ordered by the period of the first two Ca2+ peaks after TEA stimulation. Representative single-cell traces of Ca2+ oscillations (top right) and oscillation period (bottom right) in RIα KO (n = 61 cells) and WT (n = 53 cells) MIN6 β cells. ***P = 4 × 10^−4^; unpaired, two-tailed Student t test. (B) Heatmap (left) depicting temporal dynamics of cAMP oscillations in individual WT or RIα-null (RIα KO) MIN6 β cells (each row), shown as normalized cAMPFIRE C/Y emission ratios. Cell ordering reflects the period between the initial two cAMP peaks following TEA stimulation. Representative cAMP oscillation traces (top right) and period quantification (bottom right) in WT (n = 19 cells) or KO (n = 31 cells). ***P = 0.0002; unpaired, two-tailed Student t test. Data represent mean ± SEM. The data underlying this figure can be found in S1 Data. (TIF) [file pbio.3003262.s004.tif]

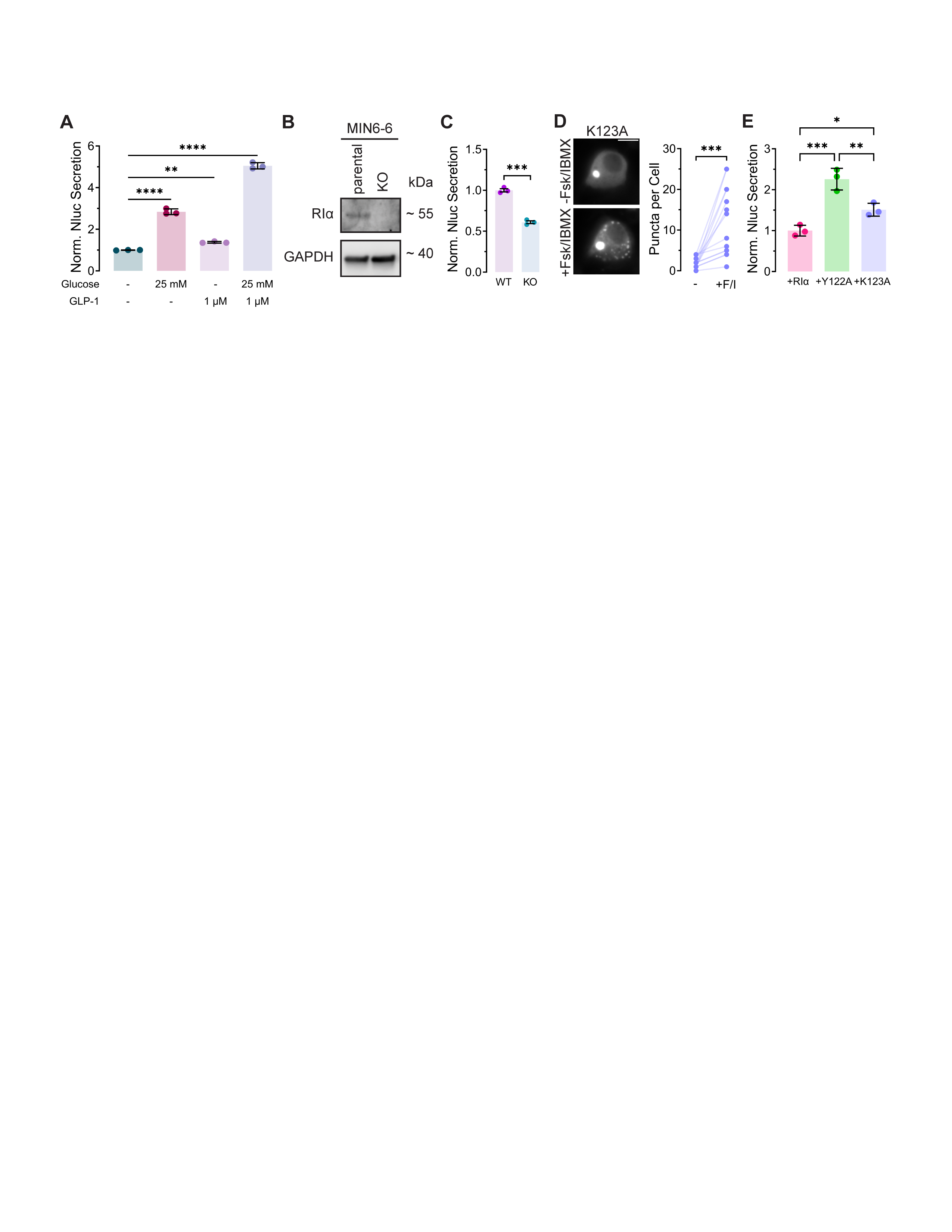

Supplement: S5 Fig — (A) Insulin secretion assay in MIN6-6 cells under different stimulation conditions. Cells were treated with glucose (25 mM), GLP-1 (1 µM), or both. (Glucose: ****P < 0.0001; GLP-1: **P < 0.0052; Glucose and GLP-1: ****P < 0.0001; n = 3 for all conditions). Statistical analysis was performed using ordinary one-way ANOVA followed by Dunnett’s multiple comparisons test. Error bars represent mean ± SEM. (B) Western blot results showing knockout of RIα in MIN6-6 β cell line. (C) Relative insulin secretion level between parent MIN6-6 and RIα-null MIN6-6 cells upon glucose (25 mM)/GLP-1(1 µM) stimulation. n = 3 for both, ***P = 0.000106; unpaired, two-tailed Student t test. Error bars indicate mean ± SEM. (D) Representative fluorescence images (left) and quantification of RIαK123A puncta number per cell before (−) and after (+) Fsk (50 µM) and IBMX (100 µM) stimulation. (n = 14 cells, ***P = 0.0005) (right). (E) Relative insulin secretion levels in RIα-null MIN6-β cells expressing RIαWT, RIαY122A, or RIαK123A (n = 3). *P = 0.0415, **P = 0.0078, ***P = 0.0005; ordinary one-way ANOVA followed by Tukey’s multiple-comparisons test. Error bars indicate mean ± SEM. The data underlying this figure can be found in S1 Data and S1 Raw Images. (TIF) [file pbio.3003262.s005.tif]

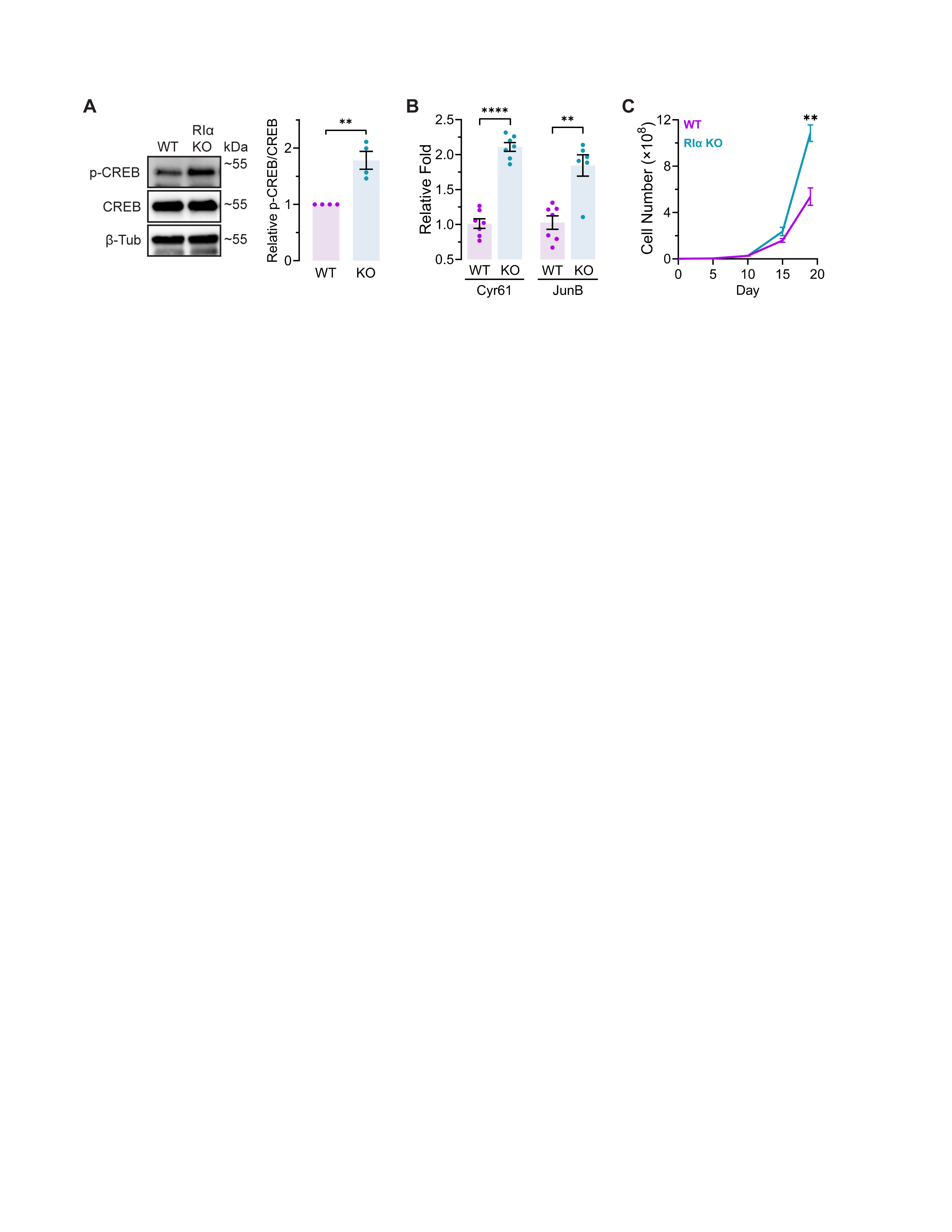

Supplement: S6 Fig — (A) Western blot of CREB and phospho-CREB (top) and quantification of p-CREB/CREB (bottom) in WT and RIα-null (KO) MIN6 β cells (n = 4 experiments). **P = 2.62 × 10^−3^; unpaired, two-tailed Student t test. (B) Quantification of Cyr61 and JunB mRNA levels in RIα KO MIN6 β cells (n = 7 experiments). ****P = 5.9 × 10^−8^; **P = 6.68 × 10^−4^; unpaired, two-tailed Student t test. (C) Cell proliferation of RIα-null (RIα KO) and wild-type (WT) MIN6 β cells (n = 4 experiments). **P = 1.82 × 10^−3^; Multiple unpaired t test. Error bars indicate mean ± SEM. The data underlying this figure can be found in S1 Data and S1 Raw Images (TIF) [file pbio.3003262.s006.tif]

**Figure 3D**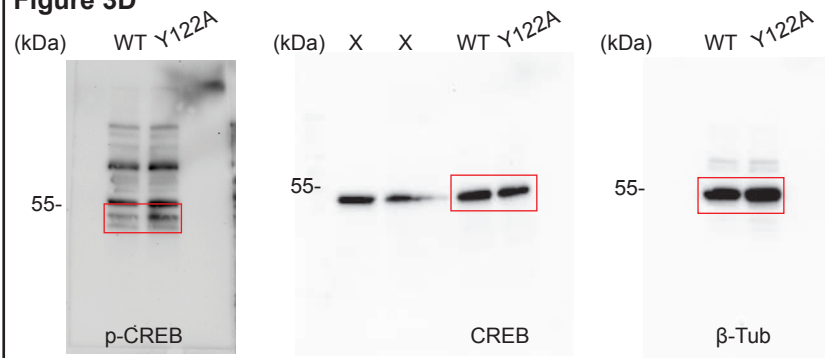**Figure S1A**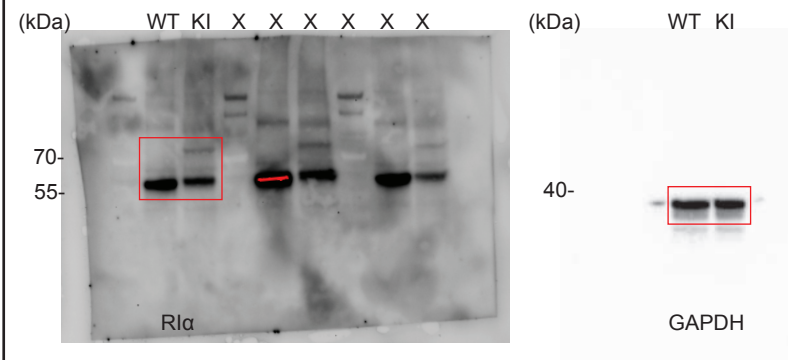**Figure S2A**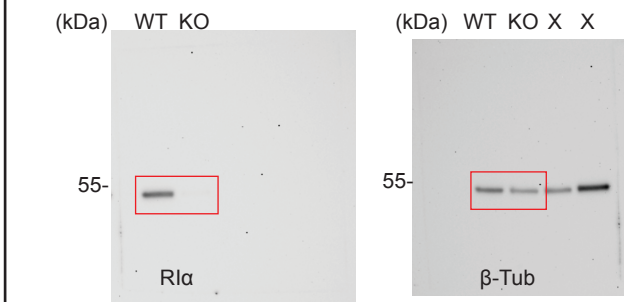**Figure S2B**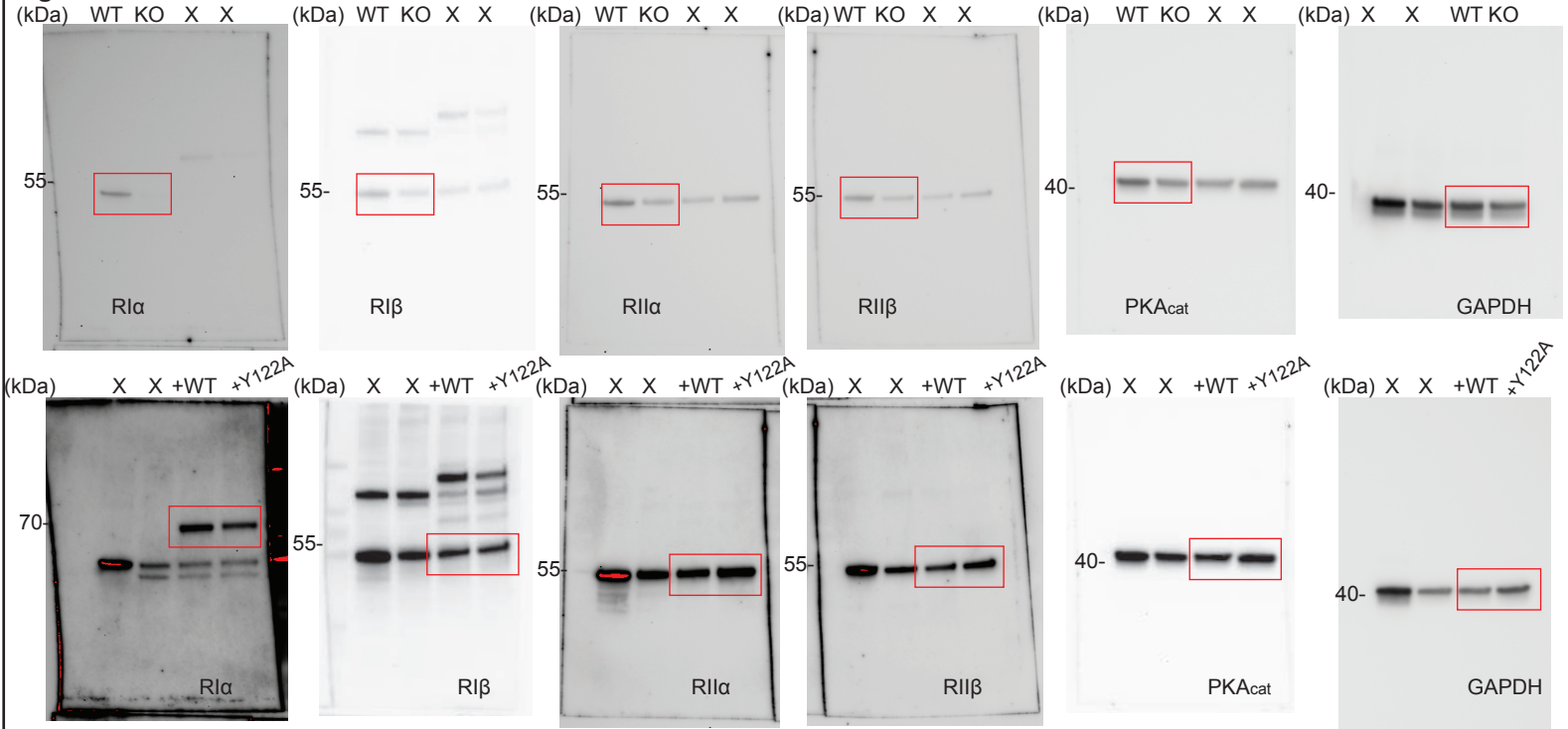**Figure S5B**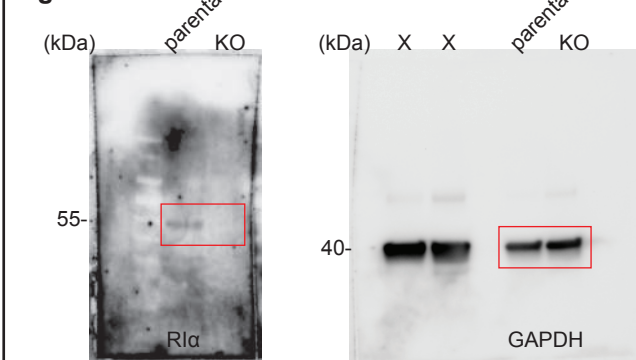**Figure S6A**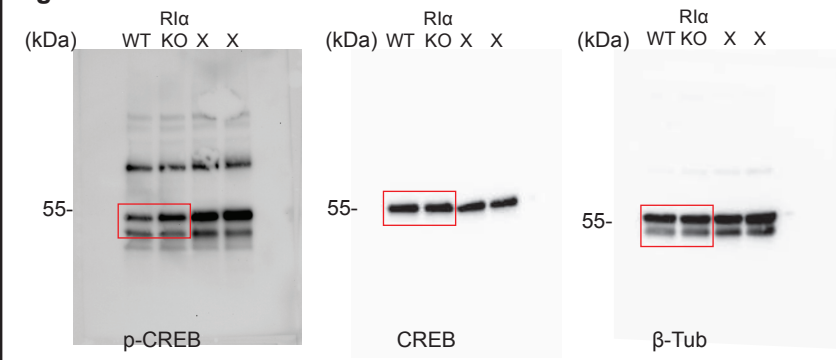

Supplement: S1 Raw Images — (PDF) [file pbio.3003262.s009.pdf]
