# Supplementary material for: Phase separation of a PKA type I regulatory subunit regulates β-cell function through cAMP compartmentalization
Source: PLoS Biol. 2025 Jul 24;23(7):e3003262. doi: 10.1371/journal.pbio.3003262 (PMC12289088; doi:10.1371/journal.pbio.3003262)
Supplement: S1 Table — (DOCX) [file pbio.3003262.s011.docx]

**Table S1** Sequences of oligonucleotides

| Purpose | Primer Name | Sequence (5’-3’) | Notes |
| --- | --- | --- | --- |
| RIα KO gRNA | KO_F | CACCGAACAACCTTCTTTCAGGAGG |  |
|  | KO_R | AAACCCTCCTGAAAGAAGGTTGTTC |  |
| RIα mutation | RIα_Y122A_F | ccaaaagatgcgaagacaatggccgctttagccaaa | Base changes are lower case |
|  | RIα_Y122A_R | gttagaaaggttataccaaaagatgcgaagacaatg | Base changes are lower case |
| RT-PCR | Cyr61_F | AAGAGGCTTCCTGTCTTTGGC |  |
|  | Cyr61_R | GTCCACAAGGACGCACTTCA |  |
|  | JunB_F | CTGGCAGCCTGTCTCTACAC |  |
|  | JunB_R | TAGCTTCAGAGATGCGCCTG |  |
|  | β-Actin_F | TAAAACCCGGCGGCGCA |  |
|  | β-Actin_R | TTCCCACCATCACACCCTGG |  |
| RIα-GFP2 knock-in | RIα_gRNA F | CACCGAGGCGCGGGTCAGACGGACA |  |
|  | RIα_gRNA R | AAACtgtccgtctgacccgcgcctC |  |
|  | 5’ homology arm F | tatacgcgttTCCTTTACTTAGTTCTGGGGCA |  |
|  | 5’ homology arm R | GTGGGGATCCgacggacagggacacgaagc |  |
|  | GFP2 F | cctgtccgtcGGATCCCCACCGGTCGCCAC |  |
|  | GFP2 R | gaggcgcgggTTACTTGTACAGCTCGTCCA |  |
|  | 3’ homology arm F | GTACAAGTAAcccgcgcctcctgtgcctcc |  |
|  | 3’ homology arm R | acagacatgaccacggttaactccccagag |  |
