# Supplementary material for: Phase separation of a PKA type I regulatory subunit regulates β-cell function through cAMP compartmentalization
Source: PLoS Biol. 2025 Jul 24;23(7):e3003262. doi: 10.1371/journal.pbio.3003262 (PMC12289088; doi:10.1371/journal.pbio.3003262)
Supplement: Attachment — Submitted filename: Response_to_PLOS_Bio_Reviewers_auresp_1.pdf [file pbio.3003262.s012.pdf]

*Reviewer #1: This is a well-executed study that takes advantage of a battery of techniques to investigate the role of LLPS of PKA type Ia subunits in MIN6 beta cells. Although the main features of Ria LLPS have been established in previous studies, this study helps to establish a wider significance to the phenomenon of RIa LLPS, and demonstrates that disrupting in RIa LLPS affects functions including gene transcription and $Ca^{2+}$ oscillations.*

We are grateful to the Reviewer for their detailed and thoughtful comments.

*Specific comments:*

*A. In the introduction (p.4): references 9-10 and 11-12 do not seem to match the text. References 9&10 are old studies that don't consider the ideal of local cAMP regulation by PDEs.*

We thank the Reviewer for this suggestion. Following the Reviewer's comment, we have cited a review paper that explains the mechanism of cAMP compartmentation, which describes the critical role of PDEs in cAMP compartmentation while suggesting that PDEs alone are not sufficient to fully explain the mechanism of cAMP compartmentation[1].

*B. On page 5, fig 1, did the authors image the blue channel to monitor C subunit puncta formation?*

We thank the Reviewer for these questions. In our original experiments, we did simultaneously monitor the blue channel, which showed that the PKA-C subunit also formed puncta together with PKA-RIα in MIN6 β cells. These data, shown below (Reviewer Figure 1), are now included in the updated Figure 1D in the revised manuscript.

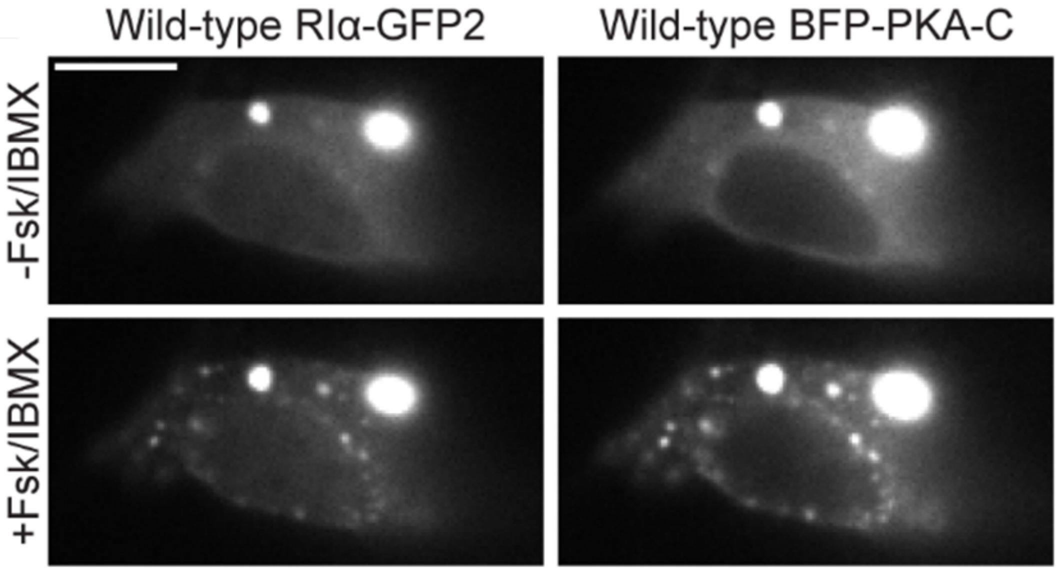


**Reviewer Figure 1. Representative fluorescence images of MIN6 β cells co-expressing RIα-GFP2 and TagBFP2-PKA-Cα.** After (+) Fsk (50 µM) and IBMX (100 µM) stimulation, RIα forms droplets and PKA-C colocalizes. Scale bar: 10µm

*C. On page 6, the manuscript would benefit from more explanation of Fig 1B - this data and the relevant numbers are not described in the main text. What exactly does 'normalised fractional recovery' mean?*

We thank the Reviewer for the opportunity to clarify. ‘Normalized fractional recovery’ refers to the degree of recovered intensity in the bleached region after it reaches steady-state. We performed two distinct data normalization steps. First, ROIs were corrected for ordinary time-course photobleaching using the average pre-bleach intensity. Experimental ROIs were then further corrected using nearby unbleached reference ROIs to account for any influence of the FRAP laser. Second, fully corrected recovery values from each experimental ROI were normalized to their minimum and maximum recorded values to generate a time course on a scale of 0 to 1, with 1 representing 100% recovery to baseline values. These data are plotted throughout the manuscript as fractional recovery. This minimum-maximum normalization allows kinetic comparison between bleaching regions with varying intensity values and is especially helpful for comparing to control experiments such as bleached bulk cytosol, which has significantly lower average starting intensity than puncta. To address the Reviewer’s concern, we simplified the term to “Fractional recovery” and amended the FRAP analysis Methods section to clarify this point:

“Intensity counts for puncta and unbleached reference puncta (one punctum and one reference punctum in the same cell, plus one cell-free background ROI constituted one sample) were background subtracted using a nearby cell-free background ROI and corrected by using the pre-bleach average intensity to account for any ordinary photobleaching over the experimental time-course. Then, corrected recovery values were normalized to unbleached reference puncta to correct for any effects of the FRAP laser. Finally, corrected recovery values for each bleached punctum were normalized to the bleached punctum time course’s minimum and maximum intensities to calculate the fractional recovery on a scale of 0 – 1, with 1.0 indicating complete recovery to baseline intensity values. (…)”

*D. On page 6, regarding the differences in speed of recovery. To what extent is the slower recovery of the pre-formed puncta likely to derive from the larger size of the pre-formed condensates? ie if you compare puncta reformation in terms of $um^2$ per unit time, do you see the same effect?*

We thank the Reviewer for raising this critical consideration in FRAP analysis. Indeed, bleaching differently sized puncta could affect the kinetics of fluorescence recovery. As the Reviewer suggested, we compared the apparent diffusion coefficients for the pre-formed large puncta and smaller newly formed condensates, calculated using the broadly used Soumpasis equation $D_{app} = 0.224r^2/t_{1/2}^2$. Consistent with the trends in half-times and mobile fractions, we found that the basal large pre-formed puncta demonstrated slower diffusion rates compared to the newly-formed puncta or regions of the bulk cytosol (Reviewer Figure 2 and Figure S1D in the revised manuscript).

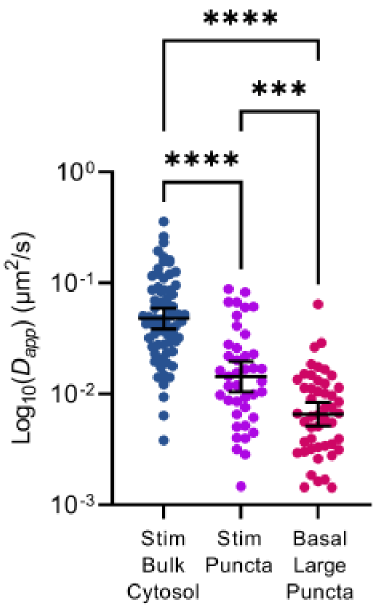


**Reviewer Figure 2. Apparent diffusion coefficients of PKA-C co-overexpressed RIα puncta in MIN6 β cells.** Error bars indicate mean ± 95% CI and statistics indicated by Brown-Forsythe and Welch ANOVA followed by Dunnett's T3 multiple comparisons test.

*E. On page 7 (Fig. 2B), several of the WT cells respond with negative FRET ratio changes upon addition of FSK, e.g., there are three data points at ~ -0.4 for WT cells. Can the authors provide an explanation for this behaviour, which is especially surprising when you consider that basal cAMP is higher in the KO cells according to Figure 2C?*

We thank the Reviewer for the opportunity to clarify. The three data points at ~ -0.4 do not represent negative responses to Fsk stimulation, but rather result from baseline drifting that was present before stimulation, which occurred in some experiments, possibly due to photobleaching. We have reanalyzed the data with baseline drift correction for these outliers and found that this does not alter our conclusions. Therefore, we have included this amended graph in the main text (Reviewer Figure 3 and Figure 2C and 2F in the revised manuscript). We have also included baseline drifting correction in the Methods.

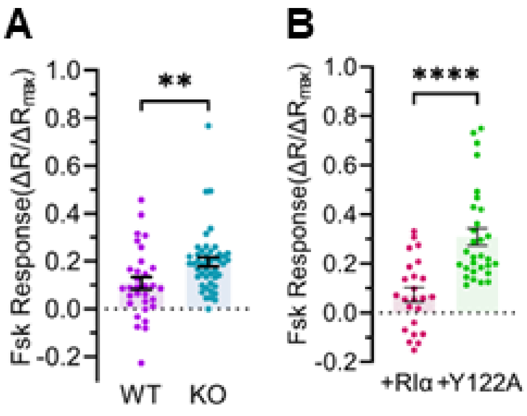

**Reviewer Figure 3. Quantification of Fsk-stimulated response in MIN6 β cells expressing PDE4D2cat-ICUE4.** Comparison of Fsk-stimulated responses between: **(A)** WT (n = 31 cells) and RIα KO cells (n = 46 cells, **P = 0.0066); **(B)** RIα KO cells expressing either $RI\alpha^{WT}$ (n = 24 cells) or $RI\alpha^{Y122A}$ (n = 30 cells, ****P = 5.17 x $10^{-6}$). Statistical analysis was performed using unpaired, two-tailed Student's t-test.

*F. Regarding the change in CREB phosphorylation (Fig 3B) - this is rather a pronounced effect. Could it be that this reflects a separation in function with RI more important for nuclear regulation vs changes at the membrane corresponding more to type II PKA? I was under the impression that $Ca^{2+}$ oscillations in beta cells would be regulated by anchored type II PKA.*

We appreciate the Reviewer's insightful comment regarding the spatial organization and functional specificity of PKA type I and II. The Reviewer's suggestion that the pronounced effect on CREB phosphorylation could reflect a spatial separation in PKA signaling is intriguing and well-reasoned, particularly given that type II PKA is known to be specifically targeted to membrane compartments through its interaction with AKAP79, which positions RII in close proximity to calcium channels for efficient regulation of calcium oscillations in β-cells[3]. Additionally, we are also considering the potential role of PDE-mediated compartmentation and changes in oscillatory dynamics in this process. In our previous work, we have shown that increased frequency of $Ca^{2+}$ and cAMP oscillations can drive elevated global signaling responses[4]. We have incorporated these perspectives in our discussion:

"This enhanced CREB phosphorylation might also reflect the distinct spatial organization of PKA signaling. While RII is known to be locally targeted to specific membrane compartments, for example through AKAP79 to regulate $Ca^{2+}$ channels and $Ca^{2+}$ oscillations[5,7,35], RI may have broader cellular effects. In addition, the increased $Ca^{2+}$ and cAMP oscillation frequency observed in these cells could also help switch PKA signaling to a global activation mode, leading to increased CREB phosphorylation[5]."

*G. A general point on protein expression levels - do the authors know the relative expression levels (ie molar ratios) of different PKA subunits in MIN6 cells? Most pertinent to this study would be the proportion of RIa vs RIbeta subunits. Furthermore, for knockdown-recovery experiments, did the authors perform western blots to compare the expression level of RI subunits in WT, KO-WT, and KO-Y122A cells?*

We appreciate the Reviewer's important questions. To address this point, we examined potential changes in PKA subunit expression, especially in RIα and RIβ levels, through Western blot analysis. We compared PKA subunit expression between MIN6 WT and RIα-null MIN6 cells, and between RIα-null-MIN6_$RI\alpha^{WT}$ and RIα-null-MIN6_$RI\alpha^{Y122A}$ cells. Apart from the expected absence of RIα expression in RIα-KO MIN6 cells, we observed no statistically significant changes (Reviewer Figure 4A and B; Figure S2B and C in the revised manuscript). Based on these results, we can reasonably speculate that the relative proportions of RIα and RIβ remained consistent throughout our experiments, particularly in studies utilizing $RI\alpha^{Y122A}$.

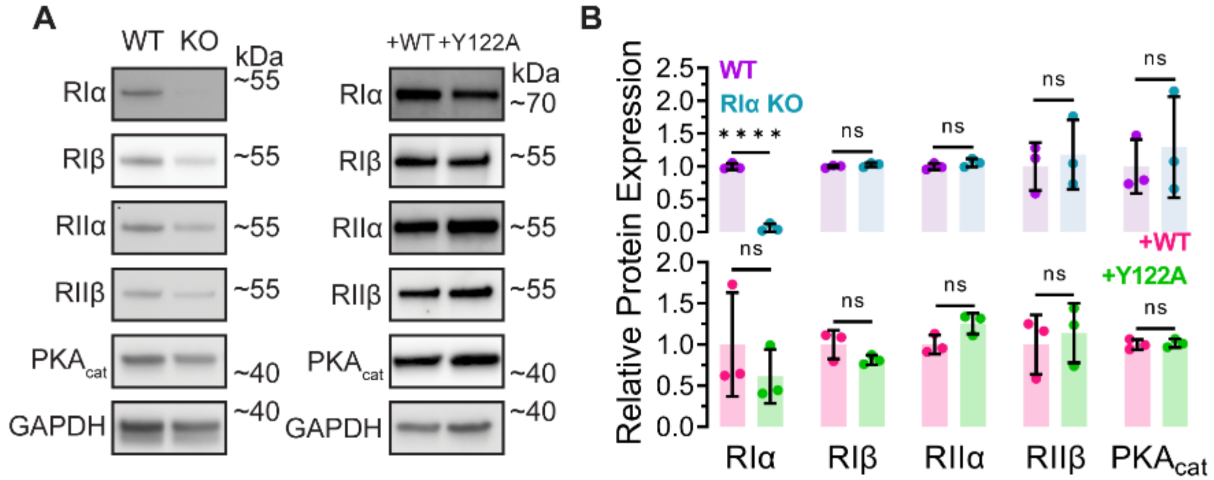

**Reviewer Figure 4. Analysis of PKA subunit expression in RIα knockout MIN6 β cells and effects of RIα$^{WT}$ or RIα$^{Y122A}$ rescue. (A)** Western blot analysis of PKA subunit expression in WT and RIα-null MIN6 β cells **(left)**, and in RIα-null cells expressing either RIα$^{WT}$ or RIα$^{Y122A}$ **(right)**. Representative blots from three independent experiments. **(B)** Quantification of Western blot data comparing WT vs. RIα-null MIN6 β cells **(top)** and RIα$^{WT}$ vs. RIα$^{Y122A}$ expression **(bottom)**. n = 3 experiments, ****P < 0.0001, unpaired two-tailed Student's t-test. Data represent mean ± SD.

*Reviewer #2: This manuscript by Lee et al demonstrates that PKA RIalpha subunits form aggregates, presumably caused by liquid-liquid phase separation (LLPS) in murine insulinoma cells. The number and size of the aggregates increase following cAMP elevation. Preventing LLPS by RIalpha knockout or expression of a mutant with reduced ability to undergo LLPS resulted in higher basal cAMP and more pronounced cAMP elevations in response to forskolin as detected by a reporter fused to PDE4D. Preventing RIalpha LLPS resulted in higher frequency of calcium oscillations, increased CREB phosphorylation and expression of Cyr61 and JunB genes, as well as an increased rate of cell proliferation. The study builds on previous work from the same laboratory demonstrating that RIalpha undergoes LLPS. The novelty largely lies in the investigation of the functional effects in a specialized cell type. The topic is timely and very interesting. The experimental tools are overall the same as in previous studies from this group but a new RIalpha mutant is introduced, which seems to hold promise to investigate LLPS in various systems. The results are clearly presented and statistical handling seems appropriate.*

We are grateful to the Reviewer for their detailed and thoughtful comment.

*The main limitation is that the study provides little insights into the physiological role of RIalpha LLPS in beta cells. These cells are specialized to sense glucose and secrete insulin and it would seem natural to investigate LLPS in relation to those fundamental properties of beta cells to support the conclusion that LLPS regulates beta cell function through cAMP compartmentation. Crucial to beta cell function is also glucagon from alpha cells, which acts via Gs-coupled receptors to increase beta cell cAMP. Rather than using tetraethylammonium and forskolin, it is therefore desirable with data on more physiological stimuli. Do e.g. glucose and glucagon (or GLP1) promote LLPS in MIN6 cells?*

We appreciate the Reviewer's suggestion. We performed a number of new experiments to address the Reviewer's questions. First, we tested GLP-1 stimulation and showed that RIα undergoes LLPS in MIN6 cells upon GLP-1 stimulation (Reviewer Figure 5 and Figure 1C). Furthermore, we performed a series of insulin secretion experiments and showed that RIα LLPS influences insulin secretion. These experimental data will be discussed below. Together these new experiments have strengthened our investigation into the physiological role of RIα LLPS. These new findings have been incorporated into the updated manuscript.

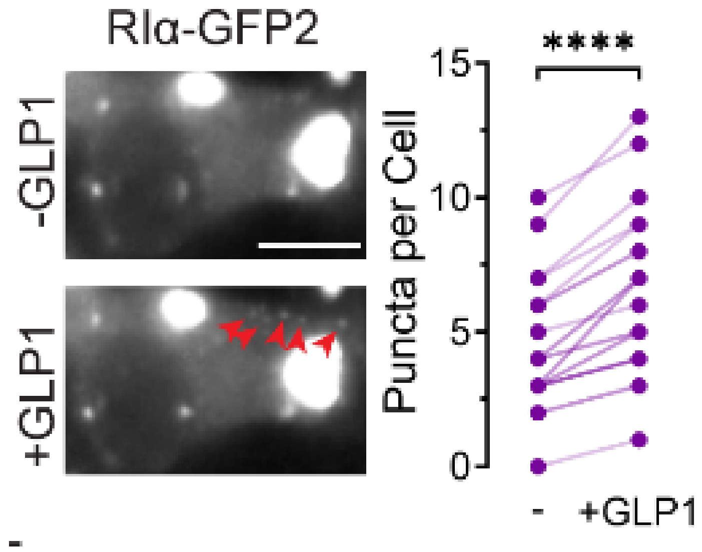


**Reviewer Figure 5. RIα undergoes LLPS upon GLP-1 stimulation.** Representative fluorescence images **(top)** and quantification of endogenous RIα puncta per cell (mean ± SEM, n = 23) in RIα-GFP2 knock-in MIN6 β cells before and after GLP-1 (1 μM) treatment **(bottom)**. ****$P<0.0001$, paired two-tailed Student's t-test. Scale bar: 10μm. Arrowheads: newly formed puncta.

*Are different cAMP levels reached in response to physiological stimuli if LLPS formation is prevented?*

We thank the Reviewer for this question. To answer this question, we measured cAMP levels using cAMPFIRE, a genetically encodable cAMP sensor, following GLP-1 stimulation in RIα-null cells expressing $RI\alpha^{WT}$ or $RI\alpha^{Y122A}$. Our results showed significantly elevated cAMP levels in MIN6 β cells expressing $RI\alpha^{Y122A}$ compared to $RI\alpha^{WT}$ (Reviewer Figure 6 and Figure 2E in the revised manuscript).

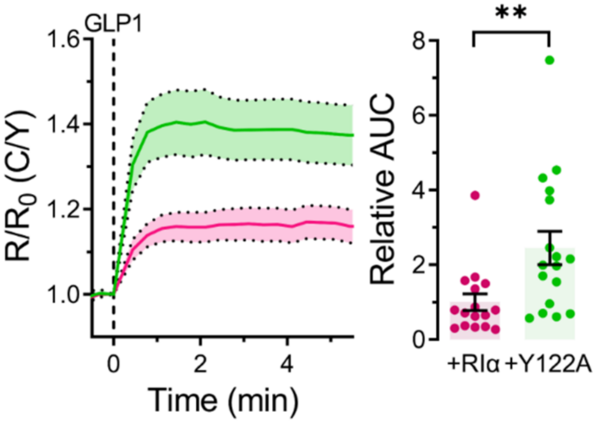

**Reviewer Figure 6. Disrupted RIα LLPS potentiates GLP-1-induced cAMP response.** Time course of normalized C/Y emission ratios of cAMPFIRE in RIα-null MIN6 β cells expressing either $RI\alpha^{WT}$ (WT) or $RI\alpha^{Y122A}$ (Y122A). Cells were stimulated with GLP-1 (1 μM) at $t_0$ (left). Area under curve (AUC) analysis from $t_0$ to $t_{5min}$, normalized to $RI\alpha^{WT}$ (right). n = 16 ($RI\alpha^{WT}$) and n = 17 ($RI\alpha^{Y122A}$). **P = 0.0079, unpaired two-tailed Student's t-test. Data represent mean ± SEM.

*Along the same lines: The readouts used here relate to proliferation, which is not a prominent feature of mature beta cells. It would be relevant to use insulin secretion as readout.*

We thank the Reviewer for this suggestion that helps enhance the impact of our research findings. We have invested considerable time and effort to address this question comprehensively. First, due to technical limitations in measuring insulin directly in our MIN6 β cell line, we utilized an engineered MIN6 cell line (MIN6-6) that enables insulin secretion measurement through luciferase activity[5]. After validating that our experimental setup could detect insulin secretion under glucose and/or GLP-1 stimulation conditions (Reviewer Figure 7A and Figure S5A in the revised manuscript), we knocked out RIα using CRISPR/Cas9 technology and confirmed this modification by Western blot (Reviewer Figure 7B and Figure S5B in the revised manuscript). Subsequently, we introduced either $RI\alpha^{WT}$ or $RI\alpha^{Y122A}$ into this cell line to compare insulin secretion. Our results showed that cells expressing $RI\alpha^{Y122A}$, which shows defective LLPS, exhibited significantly increased insulin secretion, suggesting that RIα LLPS functions to suppress insulin secretion (Reviewer Figure 7C and Figure 3C in the revised manuscript).

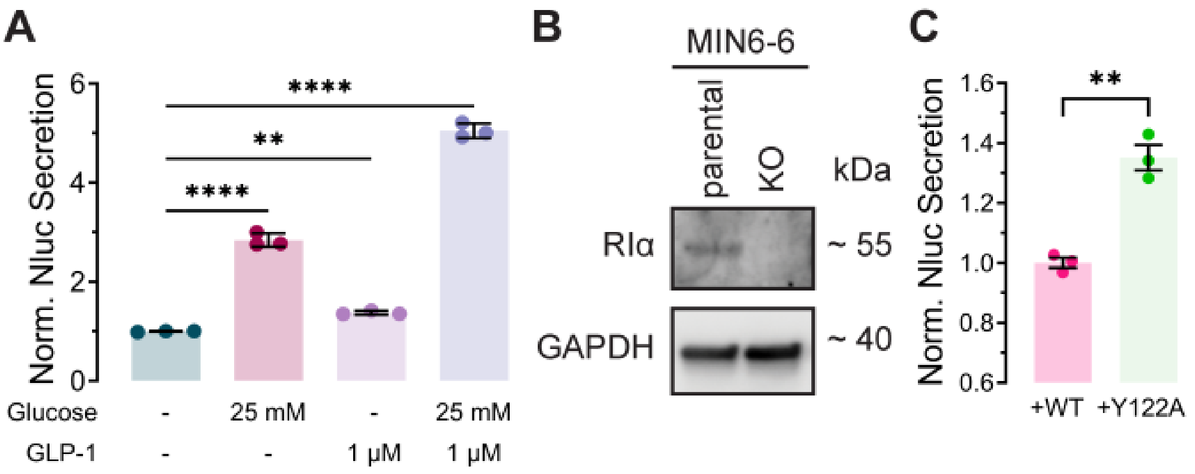


**Reviewer Figure 7. Insulin secretion assay setup and effect of RIα LLPS disruption on insulin secretion. (A)** Insulin secretion assay in MIN6-6 cells under different stimulation conditions. Cells were treated with glucose (25mM), GLP-1 (1μM), or both. (Glucose: ****P <0.0001; GLP-1: **P <0.0052; Glucose and GLP-1: ****P <0.0001; n = 3 for all conditions). Statistical analysis was performed using ordinary one-way ANOVA followed by Tukey's multiple-comparisons test. Error bars represent mean ± SEM. **(B)** Western blot confirming RIα knockout in MIN6-6 β cells. **(C)** Relative insulin secretion in RIα-null MIN6-6 cells expressing $RI\alpha^{WT}$ or $RI\alpha^{Y122A}$ upon glucose (25 mM) and GLP-1 (1 μM) stimulation. n = 3, **P = 0.0016, unpaired two-tailed Student's t-test. Data represent mean ± SEM.

*Other issues: Preventing LLPS alters calcium oscillation frequency. How is the corresponding cAMP signals altered?*

The Reviewer brings up a very interesting point. Based on our previous research showing that $Ca^{2+}$, cAMP, and PKA form an oscillatory circuit with mutual interactions[4], we anticipated that preventing RIα LLPS would affect cAMP oscillation. We tested this hypothesis through additional experiments and found that, similar to our $Ca^{2+}$ oscillation results, cAMP oscillation was also significantly accelerated in both RIα knockout cells (Figure S4B in the revised manuscript) and cells where RIα LLPS was prevented using $RI\alpha^{Y122A}$ (Reviewer Figure 8 and Figure 3B in the revised manuscript).

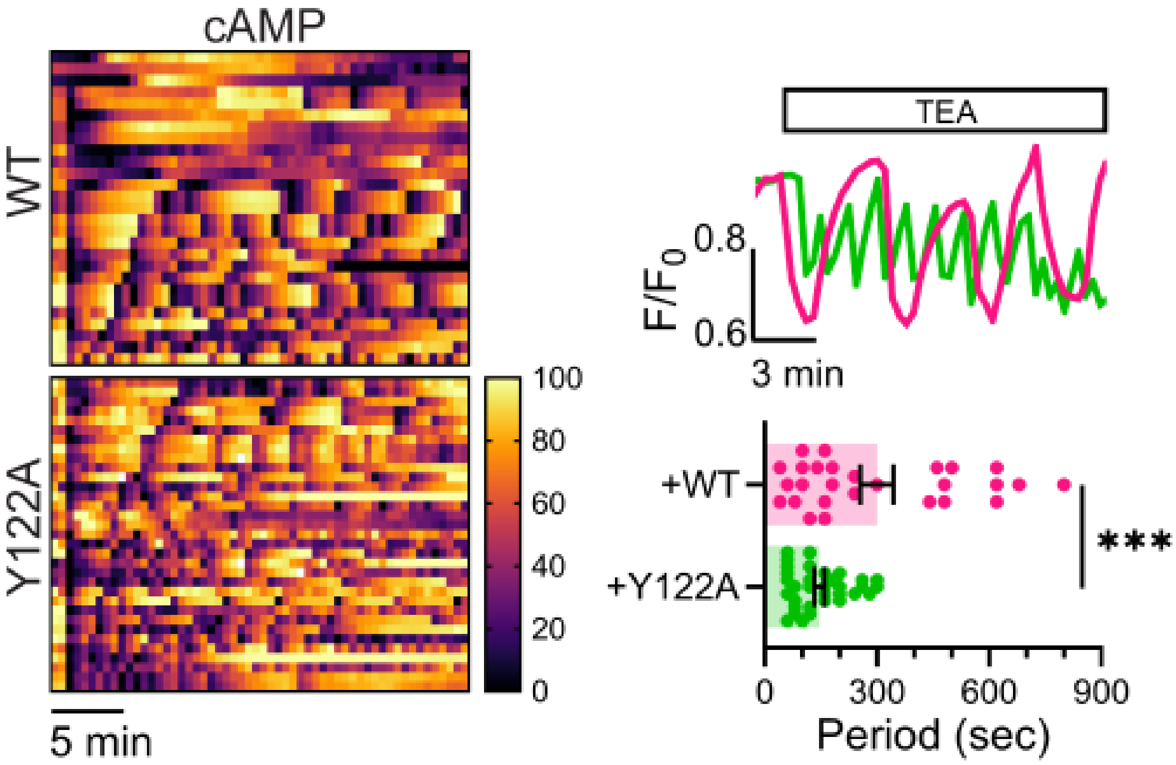


**Reviewer Figure 8. RIα LLPS disruption accelerates cAMP oscillations** Heatmap depicting temporal dynamics of cAMP oscillations in RIα-null MIN6 β cells expressing $RI\alpha^{WT}$ or $RI\alpha^{Y122A}$ as normalized cAMPFIRE C/Y emission ratios (left), with cells ordered by initial TEA-induced oscillation period. Representative traces **(top right)** and period quantification **(bottom right)** in WT (n = 33) or Y122A cells (n = 27). ***P = 0.0006, unpaired two-tailed Student's t-test. Data represent mean ± SEM.

*Is the lack of apparent difference in cAMP levels with and without LLPS during exposure to rolipram and IBMX caused by sensor saturation? It is otherwise difficult to see why levels would not differ as is the case with forskolin.*

We appreciate the Reviewer's question. The lack of apparent difference in cAMP levels with and without LLPS (RIα KO and $RI\alpha^{Y122A}$) during our sequential treatment with forskolin, rolipram (a PDE4-selective inhibitor), and IBMX (a general PDE inhibitor) could reflect several aspects. Sensor saturation at the very end is possible. In addition, under strong stimulation conditions that generate high levels of bulk cytosolic cAMP, the buffering capacity may be exceeded, making it difficult to observe significant

differences with or without buffering. Furthermore, PDE inhibition may disrupt the compartmentation itself, meaning we would no longer be measuring compartmentation effects.

*Do the cAMP levels differ also outside of the PDE nanocompartment following stimulation of cells with reduced LLPS? Only basal cAMP is measured with untethered sensor.*

We thank the Reviewer for this follow-up question. As mentioned in our previous response, we have investigated cAMP levels using cAMPFIRE, an untethered cAMP sensor, and observed significantly elevated cAMP levels in both RIα-null and $RI\alpha^{Y122A}$-expressing MIN6 cells following GLP-1 stimulation (Reviewer Figure 6 and Figure 2E in the revised manuscript). To further clarify, these measurements indeed reflect the global cAMP levels outside of the PDE nanocompartment, as we used the untethered sensor. This consistent elevation in global cAMP levels suggests that preventing RIα LLPS has a broader impact on cellular cAMP regulation beyond the local nanocompartment.

*It is not evident how dynamic LLPS buffering of cAMP is. How long do the aggregates last when stimulation ceases?*

We thank the Reviewer for this important question. To test the dynamics of RIα puncta, we induced high cAMP levels using Fsk and then ceased cAMP production using 2',3'-dideoxyadenosine (ddAdo), a selective inhibitor of transmembrane adenylyl cyclase. We observed that normalized puncta number decreased by approximately 34% within 2 minutes of ddAdo addition (Reviewer Figure 9). Notably, we observed differential dynamics between distinct populations of puncta: while pre-existing constitutive puncta showed minimal changes in number, the cAMP-responsive puncta rapidly disappeared following the cessation of stimulation. These findings suggest that LLPS-mediated cAMP buffering exhibits dynamic responsiveness, particularly in the stimulus-induced puncta population.

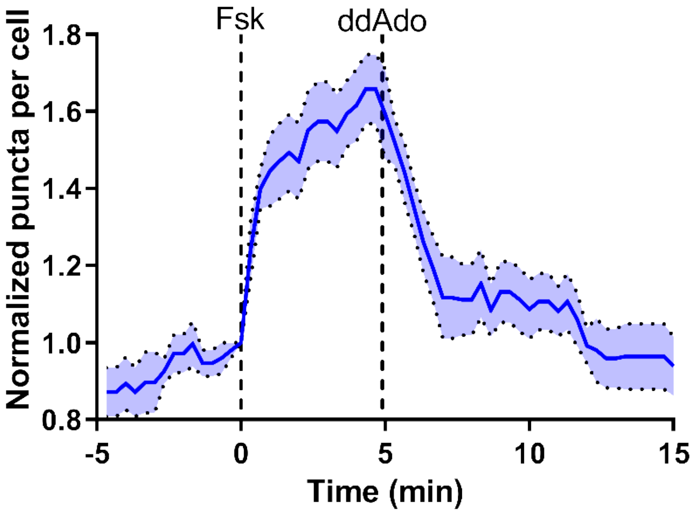


**Reviewer Figure 9. Changes in RIα Puncta after Fsk stimulation cease.** Time course of normalized RIα puncta per cell in MIN6 β cells treated with Fsk (50 μM) at $t_0$ followed by ddAdo (500 nM) at $t_{5min}$ (n = 5). Data represent mean ± SEM.

*How does RIalpha knockout affect expression of other R subunits? Would that influence cAMP buffering?*

We agree with the importance of testing whether RIα knockout affects the expression of other R subunits. We examined this using Western blot analysis, comparing not only the RIα knockout condition but also cells overexpressing $RI\alpha^{Y122A}$ with control cells. We observed no statistically significant changes for other R subunits (Reviewer Figure 10A and B; Figure S2B and C in the revised manuscript). These results support that our observed phenotypes are direct consequences of our intended RIα manipulations rather than secondary effects from altered expression of other subunits.

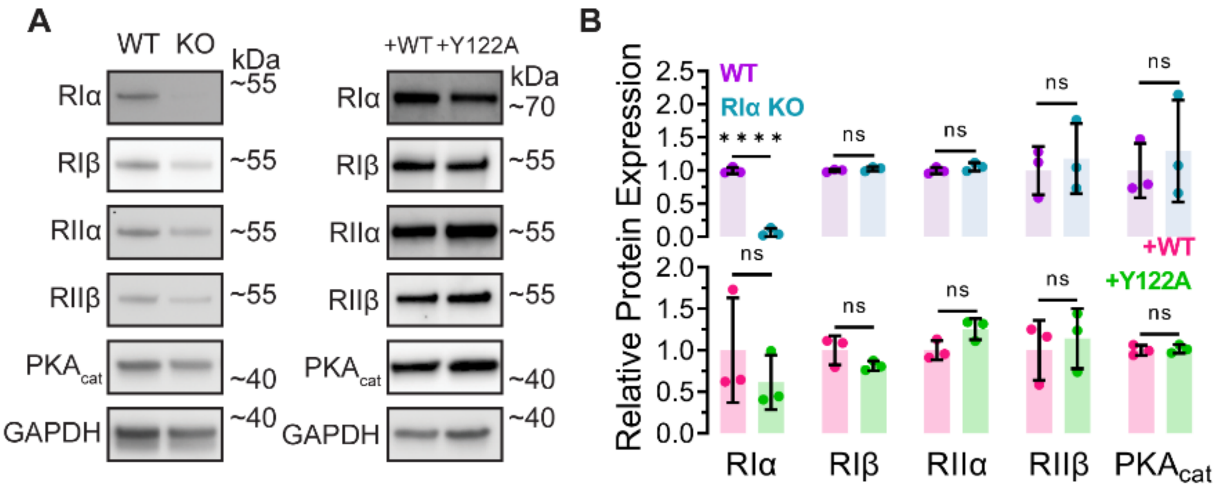


**Reviewer Figure 10. Analysis of PKA subunit expression in RIα knockout MIN6 β cells and effects of $RI\alpha^{WT}$ or $RI\alpha^{Y122A}$ rescue. (A)** Western blot analysis of PKA subunit expression in WT and RIα-null MIN6 β cells **(left)**, and in RIα-null cells expressing either $RI\alpha^{WT}$ or $RI\alpha^{Y122A}$ **(right)**. Representative blots from three independent experiments. **(B)** Quantification of Western blot data comparing WT vs. RIα-null MIN6 β cells **(top)** and $RI\alpha^{WT}$ vs. $RI\alpha^{Y122A}$ expression **(bottom)**. n = 3 experiments, ****$P < 0.0001$, unpaired two-tailed Student's t-test. Data represent mean ± SD.

*Do the puncta represent pure RIalpha or do they also contain PKA holoenzyme?*

We thank the Reviewer for this question. During our original experiments, we simultaneously monitored both the RIα and PKA-C subunits. These data confirmed that the PKA-C subunit forms puncta together with RIα upon Fsk/IBMX stimulation in MIN6 β cells and have been incorporated into the updated main figure (Reviewer Figure 11 and Figure 1D in the revised manuscript).

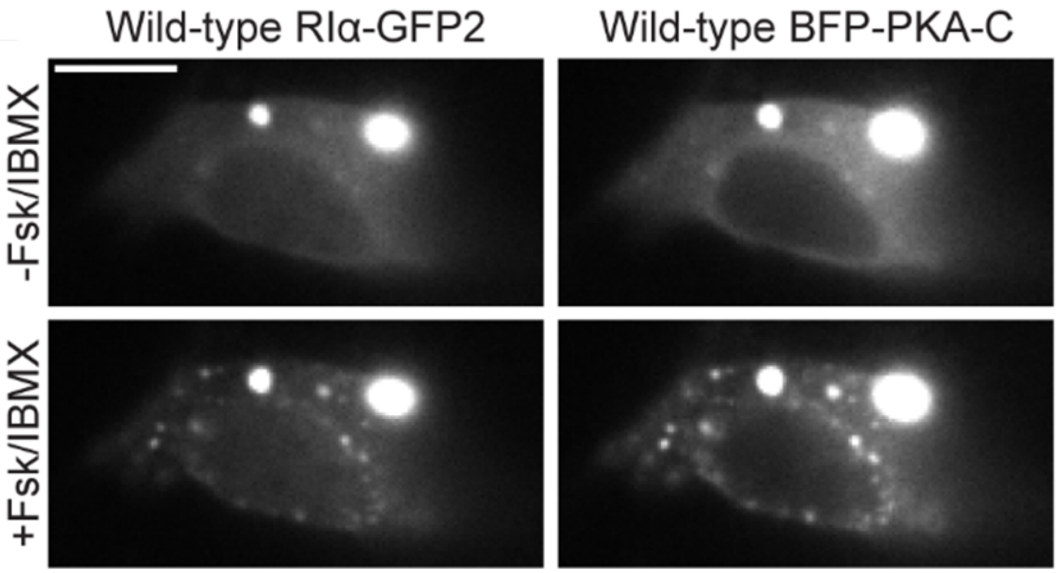


**Reviewer Figure 11. Representative fluorescence images of MIN6 β cells co-expressing RIα-GFP2 and TagBFP2-PKA-Cα.** After (+) Fsk (50 µM) and IBMX (100 µM) stimulation, RIα forms droplets and PKA-C colocalizes. Scale bar: 10µm

*Reviewer #3: In this manuscript, Lee and colleagues investigate the role of RIα liquid-liquid phase separation (LLPS) in MIN6 β cells. Previously, the same group proposed that RIα LLPS buffers cAMP, facilitating its efficient hydrolysis by phosphodiesterases and enabling cAMP signal compartmentation.*

*This reviewer is unconvinced that the data presented demonstrate cAMP buffering by RIα condensates beyond the buffering capacity provided by cAMP binding to RIα. Additionally, it is unclear whether the observed functional effects are dependent on LLPS. Essential points to be addressed:*

We sincerely thank the Reviewer for their insightful comments that help improve the quality of our manuscript. To address the Reviewer's concerns, we have conducted additional experiments as detailed below.

*1. Clear evidence that endogenous RIα can form liquid condensates is lacking. The immunostaining in Figure S1A shows agglomerates that are quite different from the puncta observed with overexpression of fluorophore-tagged RIα; they appear irregular in shape and seem to have a hollow center. In fact, these structures resemble the agglomerates formed upon overexpression of the RIαY122A mutant (Figure S2A), which the authors describe as a tool to disrupt RIα condensation. Do these agglomerates of endogenous RIα change in number or dimension with cAMP elevation? What percentage of cells exhibit these structures? Why are these structures present in MIN6 cells but not in HEK293 cells? Do these structures include Cat subunits?*

We agree with the Reviewer's concern regarding the limitations of our immunofluorescence staining using RIα antibody in demonstrating endogenous RIα LLPS. The suboptimal specificity of the RIα antibody we used made it challenging to obtain high-quality images. To overcome this limitation, we

established a MIN6 β cell line with GFP2 knocked in at the C-terminus of the RIα genomic locus using CRISPR/Cas9 technology to visualize endogenous RIα (Reviewer Figure 12A and Figure S1A in the revised manuscript). Using this approach, we confirmed that endogenous RIα forms preexisting irregular-shaped puncta in MIN6 β cells, consistent with our observations from both overexpression conditions and immunofluorescence staining (Reviewer Figure 12B and Figure 1A in the revised manuscript).

These preexisting irregular large puncta were observed in nearly all cells expressing the GFP2 knock-in construct (Reviewer Figure 12C), as well as in cells overexpressing RIα. Although the size change is not clear under endogenous RIα conditions, these large puncta exhibited quantifiable changes in size in the overexpression condition (Reviewer Figure 12D and Figure 1E in the revised manuscript). We also showed that these large puncta are more gel-like (Reviewer Figure 12E and Figure S1B in the revised manuscript) but do contain PKA-C subunits (Reviewer Figure 12F and Figure 1D in the revised manuscript).

Regarding the presence of these structures in MIN6 β cells but not in HEK293 cells, we consider this an intriguing avenue for future research. RIα is known to be in excess compared with the catalytic subunit[6]. We previously observe basal puncta in HEK293 cells, but these were round-shaped and had liquid-like properties[7]. Our current thinking is that the structures in MIN6 β cells represent gel-like condensates, formed by RIα at the basal state and "matured" over time. We will look into the possibilities of even larger excess of RIα in comparison with the catalytic subunit in MIN6 β cells versus HEK293 cells and cell type differences in heat shock proteins that are known to solubilize condensates[8] and/or in protein degradation machinery that is involved in clearing out protein aggregates[9].

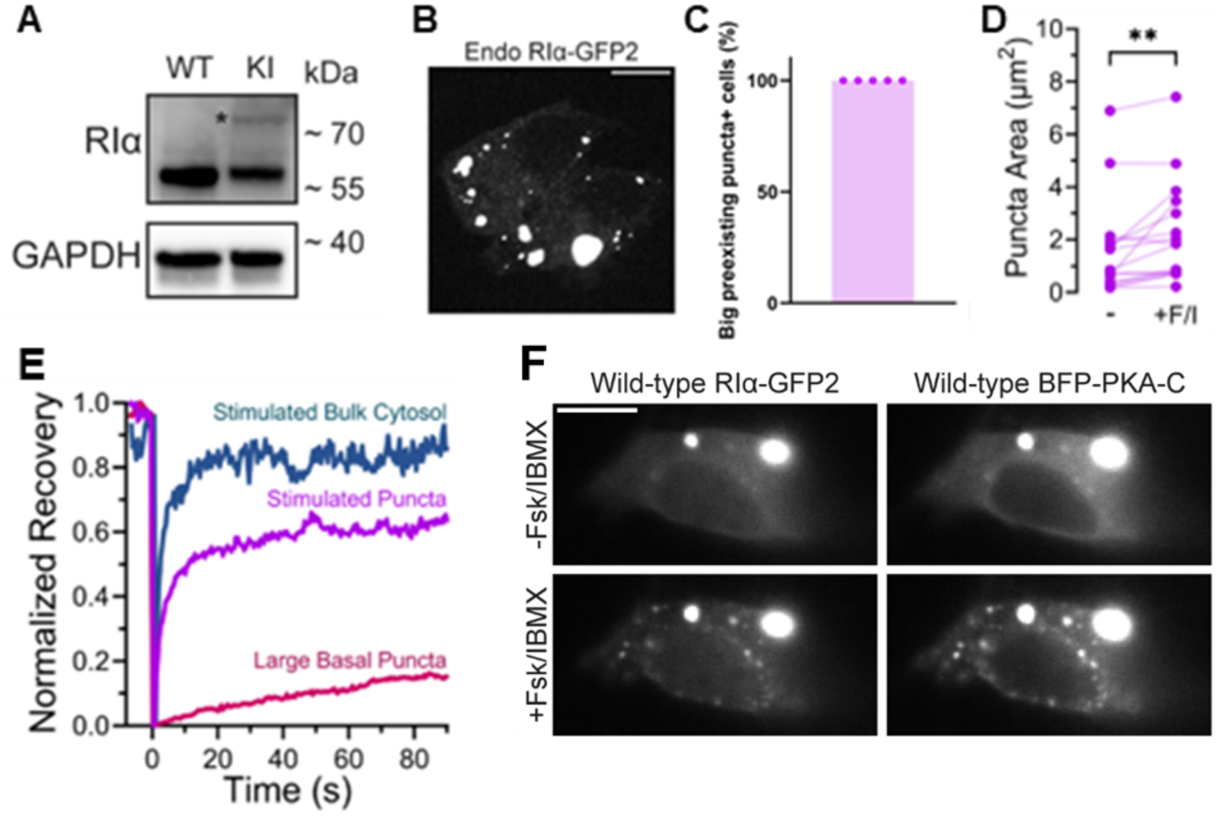

**Reviewer Figure 12. Validation of endogenous RIα LLPS and its co-phase separation with PKA-C. (A)** Western blot analysis confirming successful CRISPR-mediated homology-directed repair insertion of GFP2 into the RIα locus in MIN6 β cells. Asterisk indicates GFP2-tagged RIα. **(B)** Confocal microscopy images showing phase-separated bodies of endogenously GFP2-tagged RIα. **(C)** Percentage of GFP-positive cells containing big preexisting puncta. **(D)** Quantification of RIα puncta area (for pre-existing puncta) per cell (n = 16) in MIN6 β cells co-expressing RIα-GFP2 and TagBFP2-PKA-Cα before (-) and after (+) Fsk (50 µM) and IBMX (100 µM) stimulation. **P = 7.96 × $10^{-3}$; paired, two-tailed Student's t-test. **(E)** Representative time-courses of normalized recovery of fluorescence intensity after photobleaching of the indicated regions. **(F)** Representative fluorescence images of MIN6 β cells co-expressing RIα-GFP2 and TagBFP2-PKA-Cα before (-) and after (+) stimulation with forskolin (Fsk, 50 µM) and IBMX (100 µM). All scale bars: 10µm.

2. *The observations made in RIα knockout (KO) cells could be explained by the reduced presence of the R subunit, leading to decreased cAMP binding to R (which could account for the slightly higher basal cAMP observed in Figure 2C) and increased levels of active C subunit. This might explain the elevated calcium oscillations, CREB phosphorylation, and cell proliferation shown in Figure S3. Can this possibility be excluded? The effect of RIα KO on the expression levels of other PKA subunits (RI🚲, RII, Cat) should also be investigated, as this may influence the functional outcomes.*

We completely agree with the Reviewer's argument that RIα knockout cells might exhibit complex phenotypes due to increased levels of active C subunit, making it challenging to specifically attribute the observed cAMP changes to RIα LLPS. This is precisely why we introduced the RIα$^{Y122A}$ mutation approach as a more specific tool to investigate of the role of RIα LLPS. This mutant, which shows defective cAMP-induced LLPS but no other major signaling defects, revealed the important role of RIα LLPS in regulating $Ca^{2+}$/cAMP oscillations, insulin secretion, CREB phosphorylation, and cell proliferation.

To address the concern about the effect of RIα KO on the expression levels of other PKA subunits, we conducted comprehensive Western blot analyses to examine whether knocking out RIα or introducing RIα$^{Y122A}$ affects the expression of other PKA subunits. Our thorough analysis revealed no statistically significant changes in the expression levels of other PKA subunits (Reviewer Figure 13 and Figure S2B and C in the revised manuscript). These findings provide strong evidence that the phenotypes observed with RIα$^{Y122A}$ introduction are not due to secondary effects from altered PKA subunit expression.

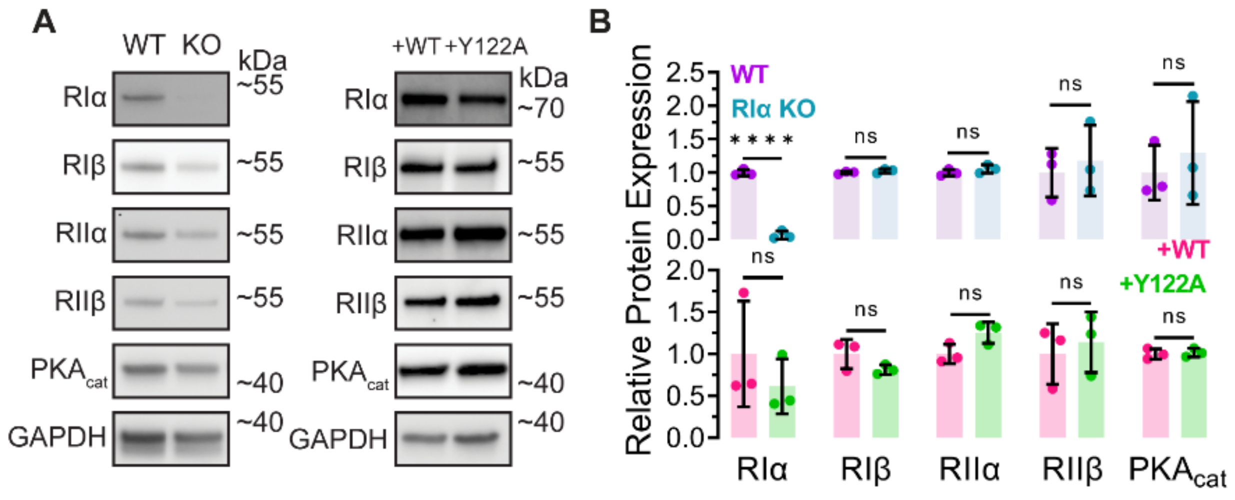

**Reviewer Figure 13. Analysis of PKA subunit expression in RIα knockout MIN6 β cells and effects of RIα$^{WT}$ or RIα$^{Y122A}$ rescue. (A)** Western blot analysis of PKA subunit expression in WT and RIα-null MIN6 β cells **(left)**, and in RIα-null cells expressing either RIα$^{WT}$ or RIα$^{Y122A}$ **(right)**. Representative blots from three independent experiments. **(B)** Quantification of Western blot data comparing WT vs. RIα-null MIN6 β cells **(top)** and RIα$^{WT}$ vs. RIα$^{Y122A}$ expression **(bottom)**. n = 3 experiments, ****$P < 0.0001$, unpaired two-tailed Student's t-test. Data represent mean ± SD.

*3. The evidence that the Y122A RIα mutant effectively disrupts LLPS is not robust. The data in Figure S2E show that this mutant forms agglomerates with a morphology quite different from that of overexpressed RIα condensates. The fact that these agglomerates do not increase in number in response to cAMP elevation does not demonstrate disruption of RI condensation. This should be demonstrated more directly, such as through an in vitro condensation experiment where mutant RIα is titrated into a WT RIα solution and the formation of condensates is observed. Additionally, the impact of overexpressing this mutant on the expression levels of other endogenous PKA subunits (RII, Cat) should be assessed. The functional effects presented in Figure 3 could be explained by the mutant's higher sensitivity to cAMP levels. What are the functional effects of expressing the Y122A mutant in WT MIN6 cells? If the Y122A mutant disrupts RI phase separation, the functional responses in WT MIN6 should be further enhanced, especially when cells are stimulated with Forskolin.*

Thank you for raising these points about RIα$^{Y122A}$. We would like to clarify that RIα$^{Y122A}$ is not a tool to disrupt wildtype RIα LLPS, but rather a variant of RIα that exhibits reduced LLPS capability. While RIα$^{Y122A}$ maintains many characteristics of wildtype RIα[10] (Reviewer Figure 14A and B and Figure S3B and C in the revised manuscript), it specifically shows impaired ability to undergo cAMP-responsive LLPS (Reviewer Figure 14C and D and Figure S3A and F in the revised manuscript). To directly assess the phase separation propensity of RIα$^{Y122A}$ compared to wildtype, we purified RIα$^{Y122A}$ and performed *in vitro* droplet assays, comparing the previously established results with those of wildtype RIα[10] (Reviewer Figure 15 and Figure S3D in the revised manuscript). Our findings indicate that RIα$^{Y122A}$ has a shifted phase diagram, supporting our characterization of its reduced LLPS capability.

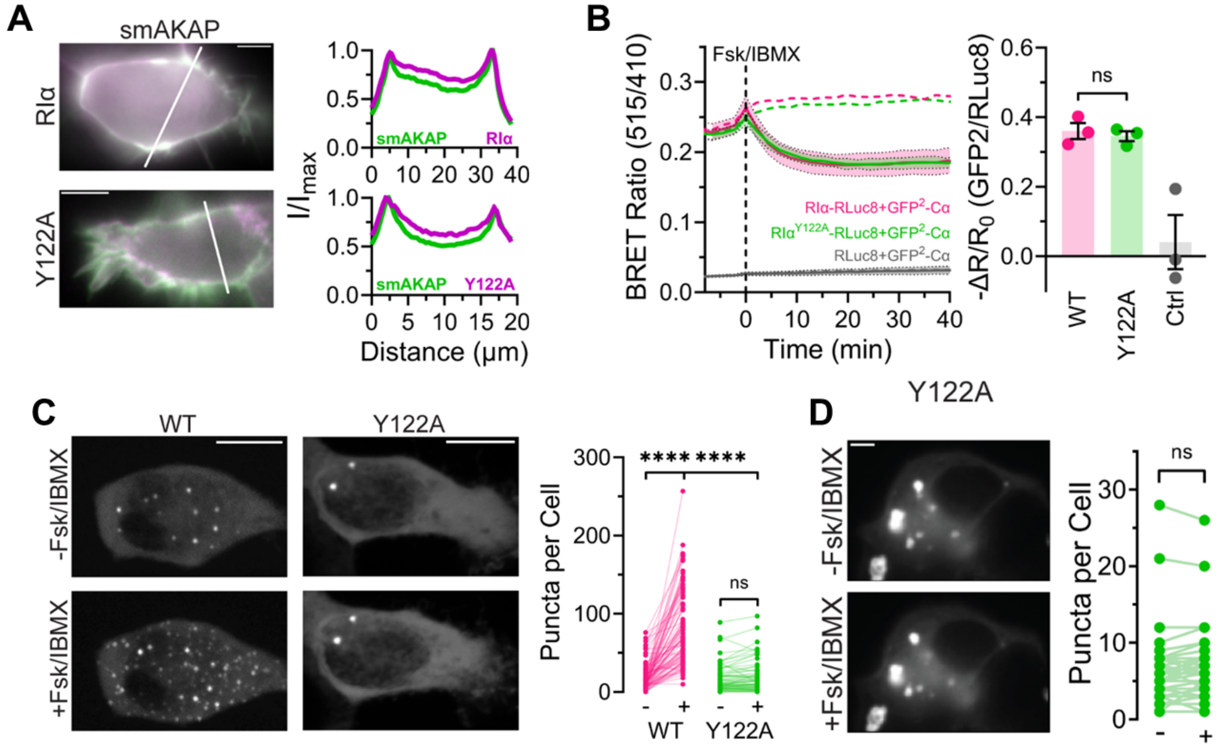


**Reviewer Figure 14. RIα$^{Y122A}$ maintains similar properties to RIα$^{WT}$ but exhibits limited LLPS formation capability. (A)** Representative fluorescent images and line-intensity profiles of the indicated regions in HEK293T cells expressing smAKAP-EGFP with mRuby2-tagged RIα$^{WT}$ or RIα$^{Y122A}$. Representative of 3 independent experiments. **(B)** BRET assay time-course (left) and quantification of Fsk-stimulated change (right) in the GFP2/RLuc8 emission ratio in HEK293T cells co-expressing GFP2-Cα plus RLuc8 fused to either RIα$^{WT}$ (n = 3 experiments) or RIα$^{Y122A}$ (n = 3 experiments). Untethered RLuc8 co-expressed with GFP2-Cα is used as a control. P = 0.61, unpaired, two-tailed Student's t-test. Solid lines indicate the mean and shaded areas indicate SEM. **(C)** Representative maximum intensity projections from confocal Z-stacks showing GFP expression (left) and quantification of puncta per cell (right) from HEK293T cells co-expressing Cα-mCherry plus GFP2-tagged RIα$^{WT}$ (WT, n = 90 cells) or RIα$^{Y122A}$ (Y122A, n = 59 cells) before (-) and after (+) Fsk and IBMX stimulation. ****$P < 1\times10^{-15}$; 2-way ANOVA with Šídák's multiple comparisons test. **(D)** Representative images (left) and quantification of puncta number (right) in RIα-null MIN6 β cells overexpressing RIα$^{Y122A}$ (n = 53 cells). ns, P = 0.17; paired, two-tailed Student's t-test. All scale bars, 10 μm.

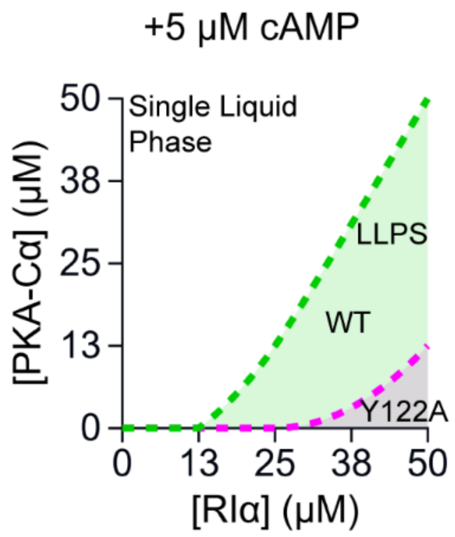


**Reviewer Figure 15. Analysis of RIα$^{Y122A}$ LLPS properties.** Critical saturation concentration (C-sat) curves of RIα$^{Y122A}$ (magenta) and RIα$^{WT}$ (green) showing PKA-Cα concentration-dependent LLPS behavior. Dotted lines show the boundary between one-phase and two-phase regions (shaded areas). RIα$^{WT}$ data were modified from our previous work[10].

Regarding the irregular structures mentioned by the Reviewer, we would like to clarify that these are not unique to RIα$^{Y122A}$, but rather a characteristic feature of RIα expression in MIN6 β cells. As we demonstrated above with our RIα-GFP2 knock-in MIN6 cells, these structures are observed with both endogenous and overexpressed RIα (Reviewer Figure 12B and F and Figure 1A and D in the revised manuscript).

Regarding the expression levels of other PKA subunits (RII, Cat), as explained in our response to the previous question, neither RIα knockout nor RIα$^{Y122A}$ introduction significantly altered the expression of other PKA subunits compared to the control (Reviewer Figure 13 and Figure S2B and C in the revised manuscript).

As for the functional effects in MIN6 β cells, we would like to emphasize again that RIα$^{Y122A}$ is not a tool to disrupt wildtype RIα LLPS, but rather a variant with impaired LLPS capability. Our experimental approach focused on introducing RIα$^{Y122A}$ into RIα-null MIN6 β cells to specifically test how the absence of RIα LLPS affects cellular function. Through these experiments, we demonstrated that when RIα cannot undergo effective cAMP-induced LLPS, the cAMP buffering effect is lost, resulting in elevated cytosolic cAMP levels under both basal and GLP-1 stimulation conditions (Reviewer Figure 16A-C and Figure 2D-F in the revised manuscript). We further showed that RIα-null cells expressing RIα$^{Y122A}$ showed faster $Ca^{2+}$/cAMP oscillations, enhanced insulin secretion, elevated CREB phosphorylation, increased CREB target gene transcription and increased cell proliferation (Reviewer Figure 17A-F and Figure 3A-F in the revised manuscript). These functional effects are unlikely due to the slightly increased sensitivity of RIα$^{Y122A}$. For example, we tested another mutant, RIα$^{K123A}$, that has the same cAMP binding characteristics as RIα$^{Y122A}$ but retains cAMP-dependent LLPS (Reviewer Figure 18A and Figure S5D in the revised manuscript). RIα$^{K123A}$ showed significantly lower insulin secretion compared to RIα$^{Y122A}$ (Reviewer Figure 18B and Figure S5E in the revised manuscript). Thus, the functional effects that we observed with RIα$^{Y122A}$

are most consistent with the loss of cAMP-induced LLPS. We have added all of these new data and also modified the description of RIα$^{Y122A}$ to avoid confusion.

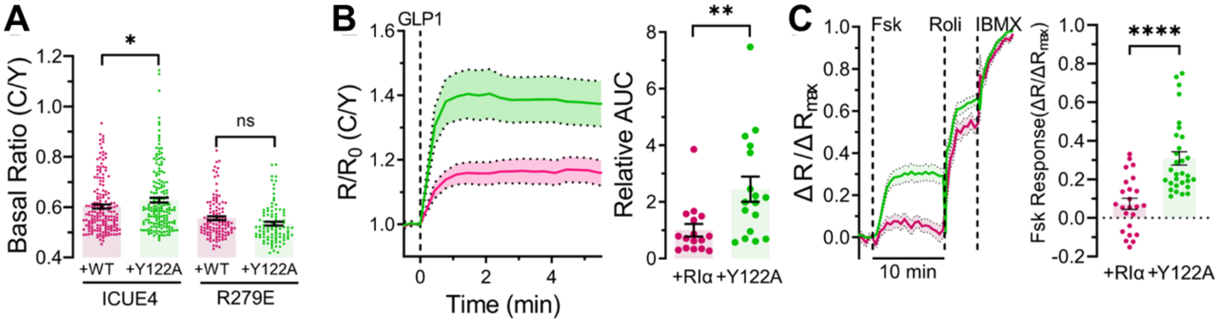


**Reviewer Figure 16. Analysis of RIα$^{Y122A}$ LLPS properties and its impact on cellular cAMP dynamics. (A)** Cyan/yellow emission ratios in RIα-null MIN6 β cells expressing RIα$^{WT}$ or RIα$^{Y122A}$ with either ICUE4 or ICUE4 (R279E). n = 113 (WT) and 96 cells (Y122A) for ICUE4; n = 170 (WT) and 177 cells (Y122A) for ICUE4 (R279E). *P = 0.043; ns, P = 0.057; unpaired, two-tailed Student's t-test. Error bars: mean ± SEM. **(B)** Time course of normalized C/Y emission ratios of cAMPFIRE in RIα-null MIN6 β cells expressing either RIα$^{WT}$ (WT) or RIα$^{Y122A}$ (Y122A). Cells were stimulated with GLP-1 (1 μM) at $t_0$ (left). Area under curve (AUC) analysis from $t_0$ to $t_{5min}$, normalized to RIα$^{WT}$ (right). n = 16 (RIα$^{WT}$) and n = 17 (RIα$^{Y122A}$). **P = 0.0079, unpaired two-tailed Student's t-test. Data represent mean ± SEM. **(C)** Time course of max-normalized emission ratio change ($\Delta R/\Delta R_{max}$) in RIα-null MIN6 β cells expressing RIα$^{WT}$ (n = 24) or RIα$^{Y122A}$ (n = 30) with PDE4D2cat-ICUE4 (left). Quantification of Fsk-stimulated response (right). Solid lines: mean; shaded areas: SEM. ****P = 5.17 x $10^{-6}$; unpaired, two-tailed Student's t-test.

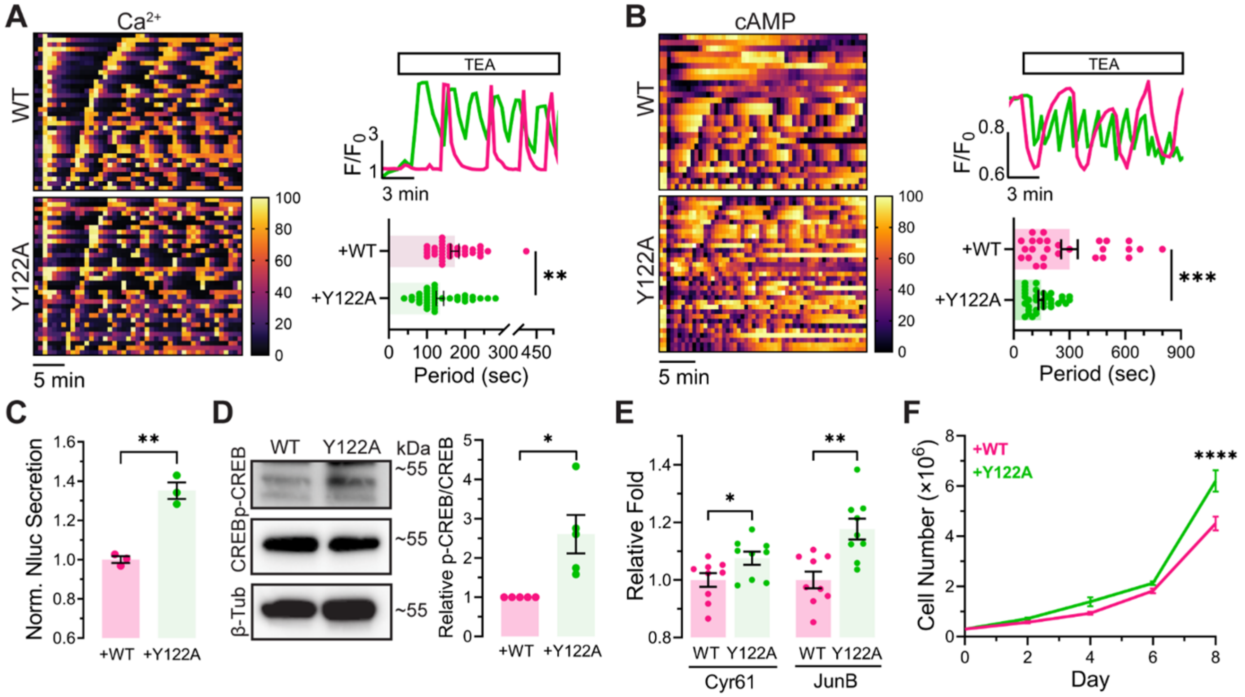

**Reviewer Figure 17. RIα LLPS regulates MIN6 β cell function. (A)** Heatmap **(left)** showing $Ca^{2+}$ oscillations in individual RIα-null MIN6 β cells (each row) expressing either $RI\alpha^{WT}$ (WT, upper) or $RI\alpha^{Y122A}$ (Y122A, lower), plotted as normalized RCaMP intensity. Cells were ordered by the period of the first two $Ca^{2+}$ peaks after TEA stimulation. Representative single-cell traces of $Ca^{2+}$ oscillation **(top right)** and quantification of oscillation period **(bottom right)** in WT (n = 34 cells) or Y122A (n = 37 cells). **P = 9.2 × $10^{-3}$; unpaired, two-tailed Student's t-test. Data represent mean ± SEM. **(B)** Heatmap **(left)** depicting temporal dynamics of cAMP oscillations in individual RIα-null MIN6 β cells (each row) expressing either $RI\alpha^{WT}$ (WT, upper) or $RI\alpha^{Y122A}$ (Y122A, lower), shown as normalized cAMPFIRE C/Y emission ratios. Cell ordering reflects the period between the initial two cAMP peaks following TEA stimulation. Representative cAMP oscillation traces **(top right)** and period quantification **(bottom right)** in WT (n = 33 cells) or Y122A (n = 27 cells). ***P = 0.0006; unpaired, two-tailed Student's t-test. Data represent mean ± SEM. **(C)** Relative insulin secretion level between in RIα-null MIN6-6 cells expressing $RI\alpha^{WT}$ (WT) or $RI\alpha^{Y122A}$ (Y122A) upon glucose (25mM) / GLP-1(1 μM) stimulation. n = 3 for both, **P = 0.0016; unpaired, two-tailed Student's t-test. Error bars indicate mean ± SEM. **(D)** Western blot of CREB and phospho-CREB (p-CREB) **(top)** and quantification of p-CREB/CREB **(bottom)** in RIα-null MIN6 β cells expressing $RI\alpha^{WT}$ (WT) or $RI\alpha^{Y122A}$ (Y122A). *P = 0.011; unpaired, two-tailed Student's t-test (n = 5 experiments). **(E)** Quantification of Cyr61 and JunB mRNA levels in RIα-null MIN6 β cells expressing $RI\alpha^{WT}$ (WT) or $RI\alpha^{Y122A}$ (Y122A). *P = 0.036; **P = 0.0016; unpaired, two-tailed Student's t-test. **(F)** Quantification of cell proliferation RIα-null MIN6 β cells expressing $RI\alpha^{WT}$ (WT) or $RI\alpha^{Y122A}$ (Y122A) (n = 4 experiments). ****P = 6.98 × $10^{-5}$; unpaired, two-tailed Student's t-test. Error bars indicate mean ± SEM.

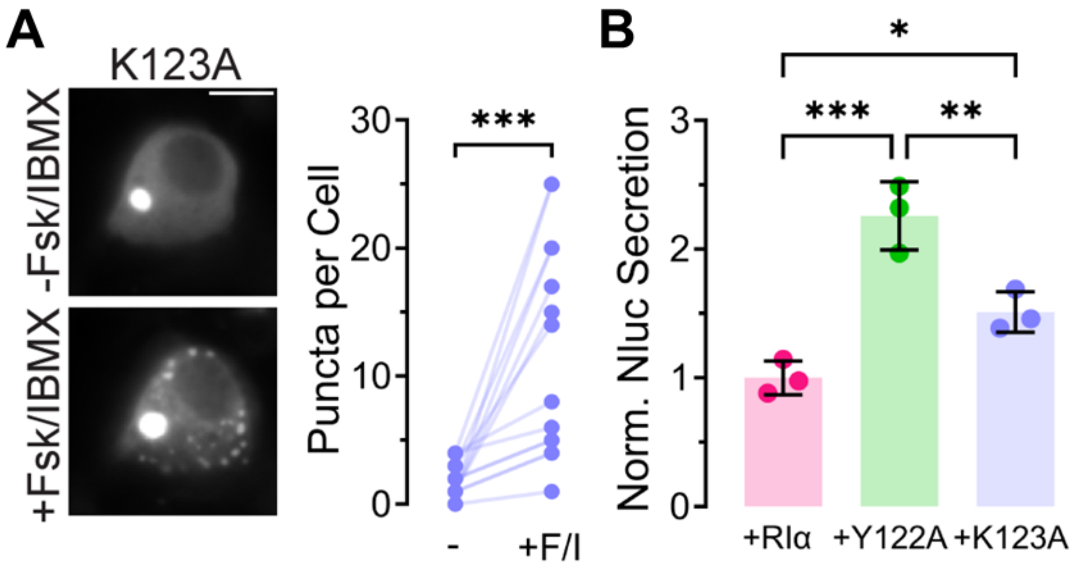


**Reviewer Figure 18. Cross-validation of $RI\alpha^{Y122A}$ effect on insulin secretion with $RI\alpha^{K123A}$. (A)** Representative fluorescence images **(left)** and quantification of $RI\alpha^{K123A}$ puncta number per cell before (-) and after (+) Fsk (50 μM) and IBMX (100 μM) stimulation **(right)**. (n = 14 cells, ***P = 0.0005) **(B)** Relative insulin secretion levels in RIα-null MIN6-6 β cells expressing $RI\alpha^{WT}$, $RI\alpha^{Y122A}$, or $RI\alpha^{K123A}$ (n = 3). **P = 0.0078, ***P = 0.0005; ordinary one-way ANOVA followed by Tukey's multiple-comparisons test. Error bars indicate mean ± SEM. Scale bar: 10μm

*4. All experiments supporting the role of RIα LLPS in cAMP compartmentation rely on detecting cAMP levels using the PDE4D2-ICUE sensor. The statement that "knocking out RIα in MIN6 β cells attenuates cAMP compartmentation" (top of page 7) is confusing, as it is unclear which compartment this specific PDE controls in these cells. Furthermore, the phosphorylation level of the PDE4D2 moiety should be assessed when the sensor is expressed in RIα KO cells and in cells expressing the Y122A mutant, as this may affect enzyme activity and explain the higher cAMP response (MacKenzie et al. J. Biol. Chem. 2000)*

We thank the reviewer for this comment that helps improve clarity for readers. We adopted the compartmentation assay from a previous study[11] and also utilized it in our previous work[7]. The "compartments" refer to nanodomains around individual PDE molecules. We acknowledge that our description of the cAMP compartmentation assay in the main text was too brief and could cause confusion. Following the reviewer's suggestion, we have expanded our explanation of the cAMP compartmentation assay and PDE4D2-ICUE sensor in the main text as follows:

"A key driver of cAMP compartmentation is the ability of PDEs degrade cAMP in their immediate vicinity, creating local cAMP sinks that act as diffusional barriers to separate distinct cAMP microdomains[6]. Our assay utilizes ICUE4 C-terminally tethered to the catalytic subunit of PDE4D2 (PDE4D2cat) to directly probe this phenomenon. Specifically, if compartmentation is intact, local cAMP degradation by PDE4D2cat will suppress the response from tethered ICUE4, whereas loss of compartmentation, such as through disrupted RIα LLPS, will rescue the ICUE4 response (Fig. S2D). Wildtype (WT) or RIα-null MIN6 β cells expressing PDE4D2cat-ICUE4 were first stimulated with Fsk to induce cAMP production, followed by the PDE4-specific inhibitor Rolipram to assess the impact of PDE4 activity on the Fsk-induced sensor response, and finally the pan-PDE inhibitor IBMX to maximally elevate cAMP and calibrate the sensor response."

Regarding the reference to MacKenzie et al. (J. Biol. Chem. 2000)[12] and the potential impact of PKA-mediated phosphorylation on PDE4 activity, we would like to clarify that we used only the catalytic domain (amino acids 86-418) of PDE4D2, which lacks the PKA phosphorylation site, to construct our sensor. This design choice was intentionally made to minimize potential confounding effects from regulatory mechanisms such as PKA-mediated phosphorylation. Therefore, while the Reviewer's concern about PKA-mediated regulation is generally valid for many PDE4 isoforms, the specific construct used in our experiments is not subject to this regulatory mechanism.

*5. The authors claim that RIα condensates act as a reservoir for cAMP and facilitate its spatial compartmentation. As in RIα KO cells this reservoir is absent, one would expect to see a larger cAMP signal in the cytosol of RIα KO cells in response to Forskolin when using untagged, cytosolic ICUE as a cAMP sensor. This signal should be reduced by the overexpression of RIα and should not be affected by the overexpression of the Y122A mutant. The conclusions would be further strengthened if the authors could demonstrate that, in the functional experiments shown in Figures 3 and S3, the effects are enhanced when cAMP is elevated by Forskolin in RIα KO cells. The response to Forskolin should be unaffected when the Y122A is expressed in RIα KO but, if the Y122A mutant disrupts puncta, the cAMP response to Forskolin should be enhanced when it is expressed in WT MIN6 cells.*

We sincerely appreciate the Reviewer's suggestions for strengthening our conclusions. Rather than using forskolin, which is a potent but non-physiological cAMP inducer, we chose to examine these effects using the more physiologically relevant GLP-1 to stimulate cAMP production. Using cAMPFIRE, a

genetically encodable sensor with higher sensitivity than ICUE4, we tested how cytosolic cAMP levels respond to GLP-1 stimulation under different conditions. Our results showed significantly elevated cAMP responses in both RIα knockout MIN6 cells (compared with wildtype cells) and RIα knockout MIN6 cells expressing $RI\alpha^{Y122A}$ (compared with RIα knockout MIN6 cells expressing $RI\alpha^{WT}$) (Reviewer Figure 19A and B; Figure 2B and E in the revised manuscript). Regarding the second suggestion that functional effects would be enhanced in situations where cAMP is elevated, we appreciate this suggestion. We therefore compared insulin secretion from RIα-knockout MIN6 cells expressing $RI\alpha^{Y122A}$ with those expressing $RI\alpha^{WT}$, both at the basal state and upon GLP-1 stimulation. While there was no significant difference in insulin secretion between the $RI\alpha^{WT}$ group and the $RI\alpha^{Y122A}$ group without GLP-1 stimulation, we observed a significant difference in insulin secretion between the two groups under GLP-1 stimulation (Reviewer Figure 20). This result is consistent with the reviewer's point that functional effects would be enhanced in situations where cAMP is elevated.

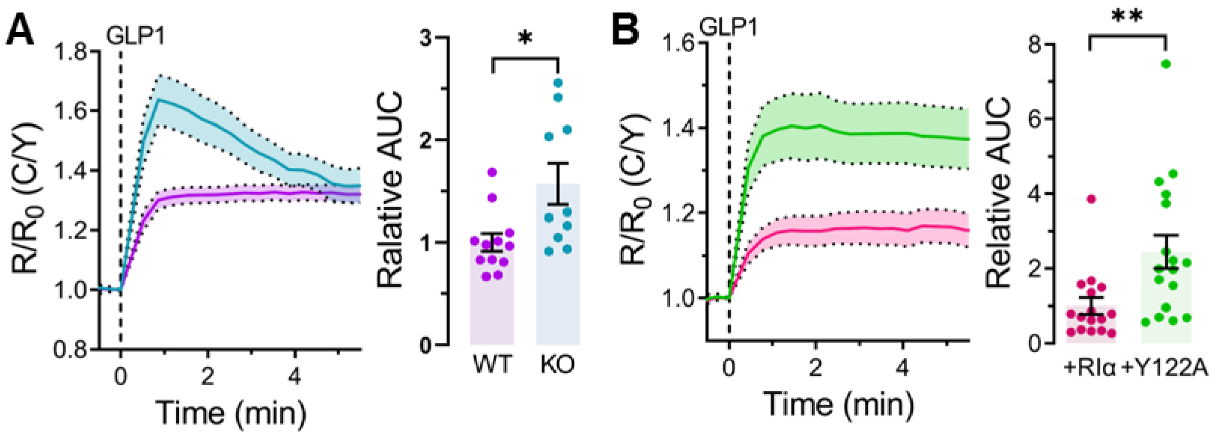


**Reviewer Figure 19. Disrupted RIα potentiates GLP-1-induced cAMP response. (A)** Time course of normalized C/Y emission ratios in WT and RIα KO MIN6 β cells expressing cAMPFIRE, showing cytosolic cAMP levels (left). Cells were stimulated with GLP-1 (1 μM) at $t_0$. Area under curve (AUC) analysis from $t_0$ to $t_{5min}$, normalized to wild-type values (right). n = 12 (WT) and n = 10 (KO). *P = 0.0116, unpaired two-tailed Student's t-test. Data represent mean ± SEM. **(B)** Time course of normalized C/Y emission ratios of cAMPFIRE in RIα-null MIN6 β cells expressing either $RI\alpha^{WT}$ (WT) or $RI\alpha^{Y122A}$ (Y122A). Cells were stimulated with GLP-1 (1 μM) at $t_0$ (left). Area under curve (AUC) analysis from $t_0$ to $t_{5min}$, normalized to $RI\alpha^{WT}$ (right). n = 16 ($RI\alpha^{WT}$) and n = 17 ($RI\alpha^{Y122A}$). **P = 0.0079, unpaired two-tailed Student's t-test. Data represent mean ± SEM.

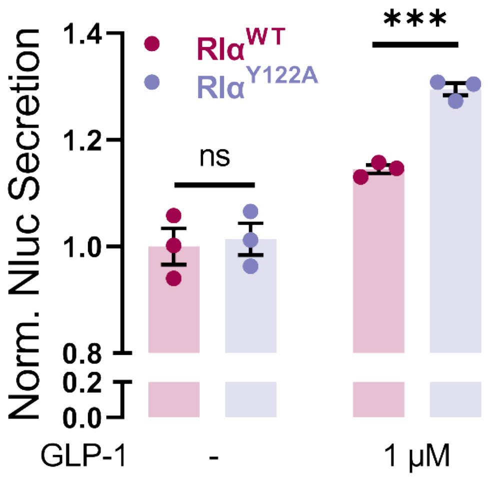


**Reviewer Figure 20. Elevated cAMP levels amplify functional differences in insulin secretion between RIα$^{WT}$ and RIα$^{Y122A}$.** Relative insulin secretion level between RIα-null MIN6-6 cells expressing either RIα$^{WT}$ or RIα$^{Y122A}$ upon with or without GLP-1(1 µM) stimulation. n = 3 for both, ***P = 0.0004; unpaired, two-tailed Student's t-test. Error bars indicate mean ± SEM.

## References


1. Saucerman, J. J., Greenwald, E. C. & Polanowska-Grabowska, R. Mechanisms of cyclic AMP compartmentation revealed by computational models. *J Gen Physiol* **143**, 39–48 (2014).

2. Soumpasis, D. M. Theoretical analysis of fluorescence photobleaching recovery experiments. *Biophys J* **41**, 95–7 (1983).

3. Murphy, J. G. *et al.* AKAP-anchored PKA maintains neuronal L-type calcium channel activity and NFAT transcriptional signaling. *Cell Rep* **7**, 1577–1588 (2014).

4. Ni, Q. *et al.* Signaling diversity of PKA achieved via a Ca2+-cAMP-PKA oscillatory circuit. *Nat Chem Biol* **7**, 34–40 (2011).

5. Yang, L. & Chen, W. Insulin secretion assays in an engineered MIN6 cell line. *MethodsX* **10**, 102029 (2023).

6. Walker-Gray, R., Stengel, F. & Gold, M. G. Mechanisms for restraining cAMP-dependent protein kinase revealed by subunit quantitation and cross-linking approaches. *Proc Natl Acad Sci U S A* **114**, 10414–10419 (2017).

7. Zhang, J. Z. *et al.* Phase Separation of a PKA Regulatory Subunit Controls cAMP Compartmentation and Oncogenic Signaling. *Cell* **182**, 1531-1544.e15 (2020).

8. Yoo, H., Bard, J. A. M., Pilipenko, E. V & Drummond, D. A. Chaperones directly and efficiently disperse stress-triggered biomolecular condensates. *Mol Cell* **82**, 741-755.e11 (2022).

9. Hao, R. *et al.* Proteasomes activate aggresome disassembly and clearance by producing unanchored ubiquitin chains. *Mol Cell* **51**, 819–28 (2013).

10. Hardy, J. C. *et al.* Molecular determinants and signaling effects of PKA RIα phase separation. *Mol Cell* **84**, 1570-1584.e7 (2024).

11. Bock, A. *et al.* Optical Mapping of cAMP Signaling at the Nanometer Scale. *Cell* **182**, 1519-1530.e17 (2020).

12. MacKenzie, S. J., Baillie, G. S., McPhee, I., Bolger, G. B. & Houslay, M. D. ERK2 mitogen-activated protein kinase binding, phosphorylation, and regulation of the PDE4D cAMP-specific phosphodiesterases. The involvement of COOH-terminal docking sites and NH2-terminal UCR regions. *J Biol Chem* **275**, 16609–17 (2000).
